# Isoforms of GPR35 have distinct extracellular N-termini that allosterically modify receptor-transducer coupling and mediate intracellular pathway bias

**DOI:** 10.1016/j.jbc.2022.102328

**Published:** 2022-08-04

**Authors:** Hannes Schihada, Thomas M. Klompstra, Laura J. Humphrys, Igor Cervenka, Shamim Dadvar, Peter Kolb, Jorge L. Ruas, Gunnar Schulte

**Affiliations:** 1 Department of Physiology and Pharmacology, Karolinska Institutet, Stockholm, Sweden; 2 Department of Pharmaceutical Chemistry, Philipps-University Marburg, Marburg, Germany; 3 Institute of Pharmacy, University of Regensburg, Regensburg, Germany

**Keywords:** G protein-coupled receptor (GPCR), cell signaling, allosteric regulation, receptor structure-function, BRET, bioluminescence resonance energy transfer, cAMP, cyclic adenosine monophosphate, DAG, diacylglycerol, FRET, fluorescence resonance energy transfer, GPCR, G protein–coupled receptor, HBSS, Hank's balanced salt solution, HEK293, human embryonic kidney 293A, NFAT-RE, nuclear factor of activated T-cells response element, Nluc, NanoLuciferase, PKN-RBD, receptor-binding domain of protein kinase N, RLUC, Renilla luciferase

## Abstract

Within the intestine, the human G protein–coupled receptor (GPCR) GPR35 is involved in oncogenic signaling, bacterial infections, and inflammatory bowel disease. GPR35 is known to be expressed as two distinct isoforms that differ only in the length of their extracellular N-termini by 31 amino acids, but detailed insights into their functional differences are lacking. Through gene expression analysis in immune and gastrointestinal cells, we show that these isoforms emerge from distinct promoter usage and alternative splicing. Additionally, we employed optical assays in living cells to thoroughly profile both GPR35 isoforms for constitutive and ligand-induced activation and signaling of 10 different heterotrimeric G proteins, ligand-induced arrestin recruitment, and receptor internalization. Our results reveal that the extended N-terminus of the long isoform limits G protein activation yet elevates receptor–β-arrestin interaction. To better understand the structural basis for this bias, we examined structural models of GPR35 and conducted experiments with mutants of both isoforms. We found that a proposed disulfide bridge between the N-terminus and extracellular loop 3, present in both isoforms, is crucial for constitutive G_13_ activation, while an additional cysteine contributed by the extended N-terminus of the long GPR35 isoform limits the extent of agonist-induced receptor–β-arrestin2 interaction. The pharmacological profiles and mechanistic insights of our study provide clues for the future design of isoform-specific GPR35 ligands that selectively modulate GPR35–transducer interactions and allow for mechanism-based therapies against, for example, inflammatory bowel disease or bacterial infections of the gastrointestinal system.

The family of G protein–coupled receptors (GPCRs) comprises more than 800 genes encoded in the human genome. These membrane-embedded receptors regulate diverse physiological and pathological processes and hence represent attractive drug targets for various diseases. In addition to the myriad of distinct GPCR-encoding genes, alternative promoter usage and pre-mRNA splicing together with alternative translation initiation add to the number of functionally distinct proteins. An exhaustive analysis of transcript-level mRNA datasets revealed that almost 40% of analyzed GPCRs have more than one isoform (1). These isoforms often exhibit tissue-specific expression patterns and differ in vital functional characteristics (2, 3, 4).

One receptor with multiple isoforms is the orphan class A (rhodopsin-like) GPCR, GPR35. The human GPR35 gene is located on chromosome 2 and can be expressed as several transcripts resulting from differential promoter usage and alternative splicing (Fig. 1*A*). All known/annotated transcripts encode the reference isoform GPR35a (hereafter referred to as ‘GPR35 short’), whereas only two mRNAs encode GPR35b (‘GPR35 long’) by alternative translation initiation. Both GPR35 variants differ only in the length of their extracellular N-termini by 31 amino acids (5). Due to the complex expression pattern of the GPR35 gene and the possibility of alternative translation initiation, there is limited information available on the tissue-specificity and activity of the short and long GPR35 variants (1).

**Figure 1:**
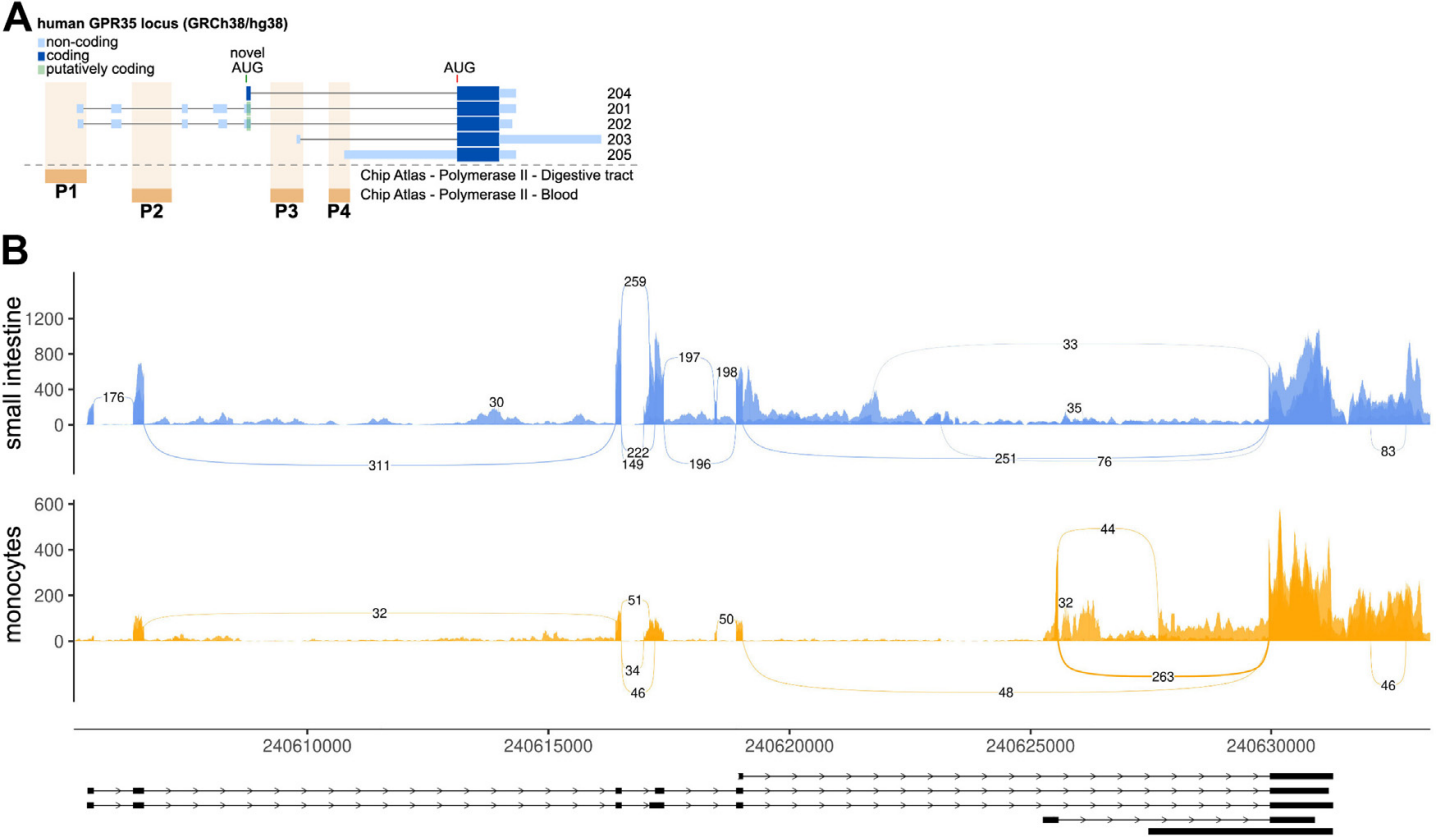
**GPR35 gene expression in human tissue.***A*, schematic representation of the human GPR35 locus. Five potential transcripts (204, 201, 202, 203, 205) and an alternative translation start site have been annotated. RNA polymerase II chromatin association sites in Chip Atlas suggest up to four gene promoters (P1-4) with the most upstream being considered canonical. *B*, sashimi plots of GPR35 splice junction analysis in human tissues (monocytes and small intestine). Numbers in splice junctions indicate the average amount of reads mapped.

GPR35 represents the verified target of the mast-cell–stabilizing, approved antiallergic drug lodoxoamide (6) and is involved in a variety of physiological processes. GPR35 controls lipid metabolism and stimulates thermogenic programs in adipocytes as well as immune cell recruitment in different situations (7, 8, 9), while its function in the intestine is associated with inflammatory bowel disease, bacterial infection, and oncogenic signaling (10, 11, 12, 13, 14, 15). In the heart, hypoxia-inducible factor-1–mediated upregulation of GPR35 expression during cardiac infarction is associated with the disruption of the actin cytoskeleton arrangement and is suggested as an early marker of progressive cardiac failure (16, 17).

Despite this crucial role of GPR35 in human health and disease, limited information is available on whether or how (patho-)physiological processes depend on specific GPR35 isoforms. While high mRNA levels of GPR35 long, but not of the short isoform, in regional lymph nodes have been suggested as a clinical marker of poor colon cancer prognosis (18), contribution of these two receptor isoforms to human pathology remains elusive. Thus far, pharmacological studies aimed at exploring the functional differences between GPR35 short and long have focused on a limited selection of receptor-mediated signaling pathways and interaction partners. Furthermore, these studies often relied on the investigation of receptor-effector fusion proteins and/or chimeric G proteins, allowing the conversion of GPR35–G protein interaction into easily detectable, albeit engineered, signaling readouts (1, 19, 20, 21, 22, 23).

In order to address these limitations, we pharmacologically characterized both GPR35 isoforms using a set of bioluminescence resonance energy transfer (BRET) and fluorescence resonance energy transfer (FRET)-based biosensors and luciferase-based reporter gene assays in human embryonic kidney 293A (HEK293) cells. To induce receptor activation, we utilized three structurally distinct agonists: (a) the partial agonist pamoic acid, which mediates the phosphorylation of extracellular signal–related kinase1/2 through a G_i/o_-dependent pathway in osteosarcoma cells (24); (b) the tryptophan metabolite kynurenic acid (21) and the full agonist zaprinast (20). Furthermore, we mapped constitutive activation of 10 distinct G protein subtypes by GPR35 isoforms using a recently established analysis scheme (25). Our data provides intriguing insights into constitutive and ligand-induced signaling profiles of GPR35 isoforms and reveals structural features of GPR35 regulating receptor-transducer coupling.

## Results

### Human GPR35 expression results from a tissue-specific combination of alternative promoter usage, splicing, and translation initiation

Among the currently annotated GPR35 transcripts (Ensemble Genome Browser, GRCh38/hg38), mRNAs 201 and 202 are expressed from the canonical, upstream promoter and contain six exons (Fig. 1*A*, P1). These transcripts are extremely similar in sequence and exon structure (with a small difference in the noncoding exon4) with the only coding exons being 5 and 6. Both transcripts can encode a long (340 aa) and a short (309 aa) GPR35 protein variant through alternative translation initiation (the two AUGs are indicated in Fig. 1*A*). Transcripts 203 and 205 are expressed from alternative downstream promoters (P3 and P4, respectively) and encode only the short GPR35 protein form. There is an additional annotated transcript (204) that consists of exons 5 and 6 only, but there is no evidence of any promoter that could drive the expression of such transcript (from, for example, chromatin accessibility or RNA polII chromatin immunoprecipitation–sequencing data; Fig. 1*B*). Moreover, the transcript 204 has no 5′ UTR present. Leaderless mRNAs are rare in higher eukaryotes and restricted mostly to mitochondrial mRNA, so it is possible for it to be an incomplete annotation of mRNAs 201/202. In any case, transcript 204 would also encode both long and short forms of GPR35. There is also a putative promoter P2 but no assignable transcript among the annotated.

To determine if GPR35 mRNAs are differentially expressed in different tissues, we analyzed RNA-seq data from monocytes and small intestine (selected for having the highest reported GPR35 expression levels). Sashimi plots showing the frequency of splicing options detected in the transcriptomes of each tissue (Fig. 1*B*) show that in the small intestine, GPR35 is preferentially transcribed from the canonical P1 promoter. This can give rise to mRNAs 201 and 202, which can encode for both long and short GPR35 forms. Conversely, monocytes seem to use less the canonical promoter and thus express mainly mRNAs 203 and 205, which encode only the short GPR35 protein.

### Surface expression of GPR35 isoforms and outline of the pharmacological profiling

**Figure 2:**
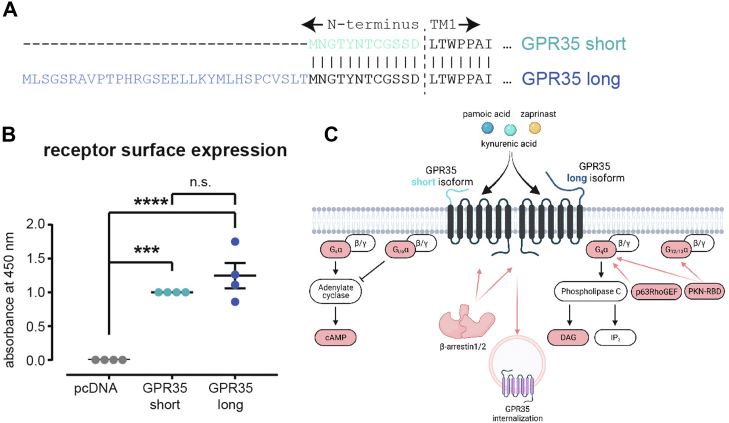
**GPR35 surface expression and project overview.***A*, sequence alignment of the variable, N-terminal regions of GPR35 short and long. *B*, comparison of receptor expression of both GPR35 isoforms at the surface of HEK293 cells. Data shown present mean ± s.e.m. of four independent experiments. Statistical significance was tested using One-way ANOVA followed by Tukey’s multiple comparison; *p* < 0.05. ∗∗∗*p* < 0.001; ∗∗∗∗*p* < 0.0001. The negative control (pcDNA transfection) was normalized to 0, and the expression of GPR35 short was set to 1.0 for each individual experiment. *C*, overview of the employed GPR35 ligands and intracellular signaling pathways monitored in the present study. The signaling components highlighted in *red* were detected in living cells using different optical biosensors. HEK293, human embryonic kidney 293A.

Prior to pharmacologically characterizing the GPR35 isoforms, we first quantified the expression levels of N-terminally FLAG-tagged GPR35 short and long, which only differ by a 31 amino acid long extension (Fig. 2*A*), on the surface of HEK293A cells by performing ELISA experiments with intact cells (Figs. 2*B* and S1). Both isoforms showed distinct expression levels and we did not detect any differences in surface expression between the two isoforms, simplifying the interpretation of potential differences in E_max_ and/or EC_50_ in subsequent signaling experiments. Figure 2*C* summarizes the signaling events that we monitored in this study using a diverse toolbox of optical biosensors.

First, we analyzed constitutive and ligand-induced G protein activation mediated by GPR35 short and long. In contrast to previous studies, we used BRET-based, tricistronic G protein activity sensors, which report G protein activation as a decrease in BRET (25), coexpressed with either isoform of GPR35. To extend our panel of eight G protein subtype-specific biosensors, we employed the same sensor design based on the insertion of NanoLuciferase (Nluc) and a yellow fluorescent protein (cpVenus) to engineer probes for pertussis toxin-insensitive G_z_ and G_12_. The fidelity of these new G protein sensors was confirmed with previously validated G_12_- and G_z_-coupled receptors, the thromboxane A_2_ receptor, and the histamine H_3_ receptor, respectively (Fig. S2).

### Constitutive G protein activation by GPR35

**Figure 3:**
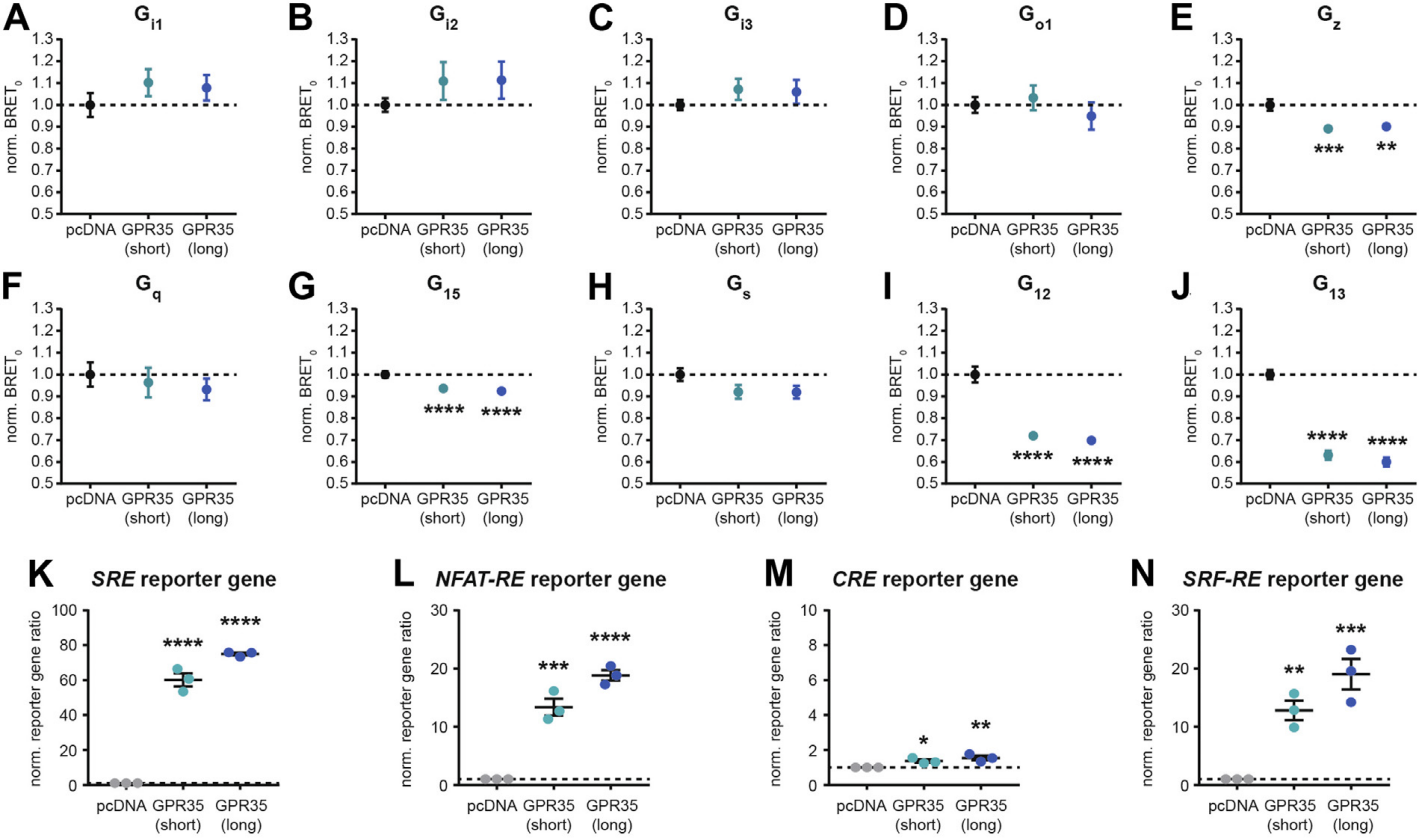
**Constitutive activation of heterotrimeric G proteins by GPR35.***A–J*, normalized BRET_0_ values of the indicated G protein sensors cotransfected with pcDNA or GPR35 isoforms. *K–N*, transcriptional activity in cells cotransfected with G protein–dependent reporter gene plasmids along with pcDNA or GPR35 isoforms. Data show mean ± s.e.m. of nine (*A–J*) or three (*K–N*) independent experiments conducted in transiently transfected HEK293A cells. Statistical significance was tested using One-way ANOVA followed by Tukey’s multiple comparison; ∗*p* < 0.05; ∗∗*p* < 0.01; ∗∗∗*p* < 0.001; ∗∗∗∗*p* < 0.0001. BRET, bioluminescence resonance energy transfer; HEK293, human embryonic kidney 293A.

Because constitutive, ligand-independent signaling of GPR35 has already been suggested following the initial cloning of this receptor (5), but has, thus far, not been thoroughly characterized across all G protein families, we first set out to quantify constitutive activation of heterotrimeric G protein biosensors (Figs. 3, *A*–*J* and S3). Employing the biophysical BRET_0_ approach (25), we found that both GPR35 isoforms constitutively activate G_z_, G_15_, G_12_, and G_13_—with the strongest effect observed for the G_12/13_ protein family (Fig. 3, *E*, *G*, *I,* and *J*). Constitutive activation of G_i/o_, G_q_, and G_s_ proteins, however, could not be detected. Collectively, constitutive activation of these G proteins resulted in substantially elevated activity of serum response element (primarily downstream of active G_i/o_ proteins), nuclear factor of activated T-cells response element (*NFAT-RE*, primarily downstream of G_q/11_ proteins including G_15_), and serum response factor response element (primarily downstream of G_12/13_), whereas transcription of the cyclic adenosine monophosphate (cAMP) response element (primarily downstream of G_s_) was only slightly increased and could likely be a consequence of signaling crosstalk (Fig. 3, *K*–*N*).

### Effect of agonist stimulation on GPR35-mediated G protein activation and signaling

**Figure 4:**
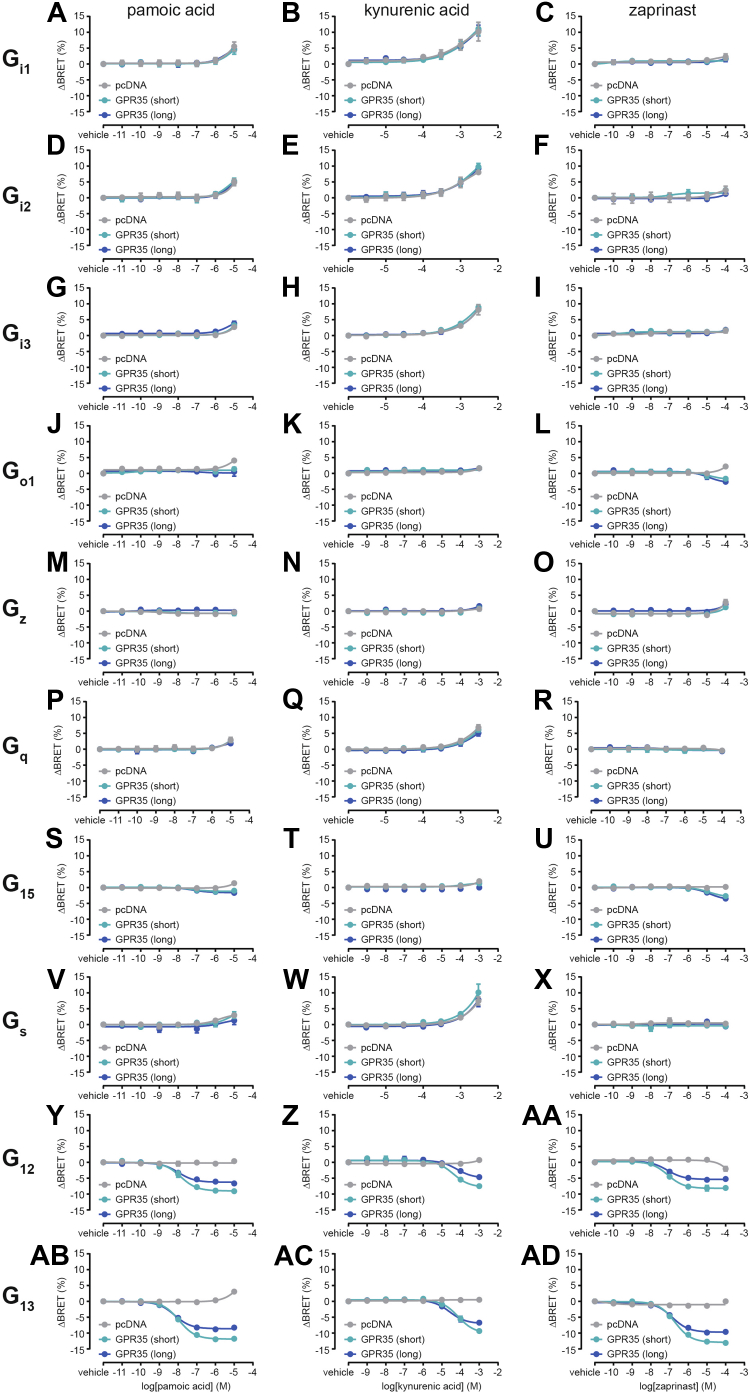
**Ligand-induced, GPR35-mediated activation of heterotrimeric G protein biosensors.** Concentration response curves of pamoic acid, kynurenic acid, and zaprinast obtained using BRET-based biosensors of G_i1_ (*A*–*C*), G_i2_ (*D*–*F*), G_i3_ (*G*–*I*), G_o1_ (*J*–*L*), G_z_ (*M*–*O*), G_q_ (*P*–*R*), G_15_ (*S*–*U*), G_s_ (*V*–*X*), G_12_ (*y*–*aa*), and G_13_ (*ab*–*ad*). Data show mean ± s.e.m. of three to four independent experiments conducted in transiently transfected HEK293A cells. Vehicle-treated ΔBRET values were set to 0. BRET, bioluminescence resonance energy transfer; HEK293, human embryonic kidney 293A.

Using the same set of biosensors, we next set out to investigate G protein activation upon stimulation of GPR35 isoforms with pamoic acid, kynurenic acid, or zaprinast (Figs. 4, S4, and S6, Tables S1–S3). Our experiments revealed that neither GPR35 isoform is capable of activating G_i1_, G_i2_, G_i3_, G_q_, or G_s_ in the presence of agonist and that agonist binding to GPR35 does not promote further activation of G_z_ (Fig. 4, *A*–*I*, *M*–*R*, *V*–*X* and Tables S1–S3). Minor differences between pcDNA control- and GPR35-transfected cells with G_o1_ and G_15_ biosensors following stimulation with pamoic acid or zaprinast did not result in statistical significance (Fig. 4, *J*, *L*, *S*, *U*, and Tables S1–S3). In contrast, substantial activation of G_12_ and G_13_ was induced upon stimulation with any of the three agonists (Fig. 4, *y*–*ad*). While only slight, yet statistically significant, increases in agonist potency to mediate G_13_ activation *via* GPR35 long could be observed, substantially different BRET maxima were measured for activated G_12_ and G_13_ biosensors: agonist stimulation of GPR35 short consistently induced a 30 to 40% larger decrease in BRET than GPR35 long (Tables S1–S3).

**Figure 5:**
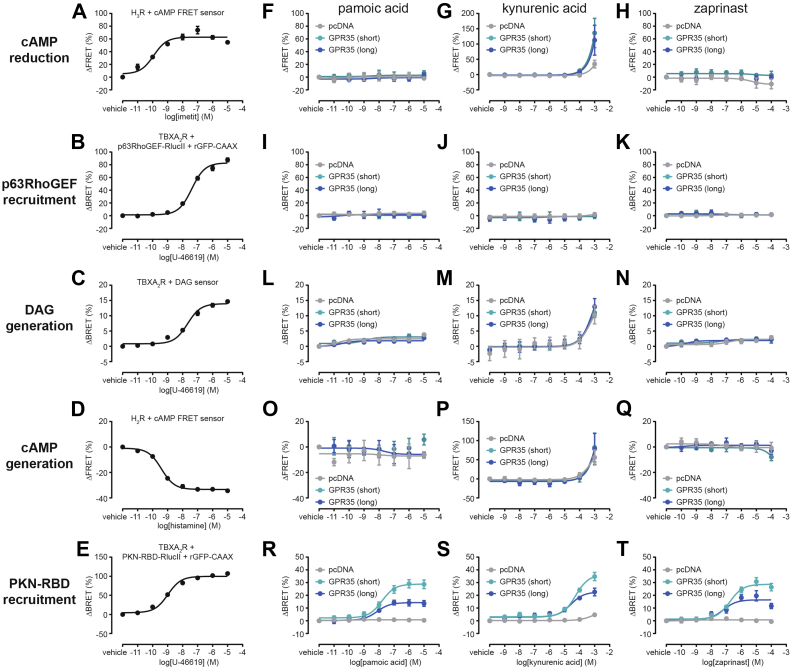
**GPR35-mediated intracellular signaling.***A*, reduction of forskolin-induced cAMP levels elicited by imetit in histamine H_3_ receptor (H_3_R)-transfected cells. *B*, recruitment of p63RhoGEF-RlucII to the plasma membrane upon activation of thromboxane A_2_ receptors (TBXA_2_R) with U-46619. *C*, generation of DAG upon activation of TBXA_2_R with U-46619. *D*, generation of cAMP upon activation of histamine H_2_ receptors (H_2_R) with histamine. *E*, recruitment of the receptor-binding domain of protein kinase N (PKN-RBD) to the plasma membrane upon activation of TBXA_2_R with U-46619. *F*–*H*, reduction of forskolin-induced cAMP second messenger levels upon stimulation of pcDNA-, GPR35 short-, or GPR35 long-transfected cells with pamoic acid (*F*), kynurenic acid (*G*), or zaprinast (*H*). *I*–*K*, recruitment of p63RhoGEF-RlucII to the plasma membrane upon stimulation of pcDNA-, GPR35 short-, or GPR35 long-transfected cells with pamoic acid (*I*), kynurenic acid (*J*), or zaprinast (*K*). *L*–*N*, generation of DAG upon stimulation of pcDNA-, GPR35 short-, or GPR35 long-transfected cells with pamoic acid (*L*), kynurenic acid (*M*), or zaprinast (*N*). *O*–*Q*, generation of cAMP upon stimulation of pcDNA-, GPR35 short-, or GPR35 long-transfected cells with pamoic acid (*O*), kynurenic acid (*P*), or zaprinast (*Q*). *R*–*T*, PKN-RBD recruitment to the plasma membrane upon stimulation of pcDNA-, GPR35 short-, or GPR35 long-transfected cells with pamoic acid (*R*), kynurenic acid (*S*), or zaprinast (*T*). Data show mean ± s.e.m. of three to four independent experiments conducted in transiently transfected HEK293A cells. Vehicle-treated ΔBRET and ΔFRET values were set to 0. DAG, diacylglycerol; PKN-RBD, receptor-binding domain of protein kinase N; BRET, bioluminescence resonance energy transfer; HEK293, human embryonic kidney 293A.

To confirm these results obtained with the G protein biosensors, we employed readouts of signaling events downstream of activated heterotrimeric G proteins. To detect G_i/o_- and G_s_-mediated signaling, we preincubated cells with either an activator of adenylyl cyclases forskolin (5 μM) or with the phosphodiesterase inhibitor 3-isobutyl-1-methylxanthin (5 μM), respectively, and assessed ligand-induced changes in intracellular cAMP levels with the FRET biosensor H187 (26). Additionally, we monitored membrane recruitment of Rho-specific guanine nucleotide exchange factor p63RhoGEF and generation of diacylglycerol (DAG)—two independent processes mediated by active G_q/11_—as well as translocation of the receptor-binding domain of protein kinase N (PKN-RBD) to the cell surface, which is promoted by active G_12/13_, with established bystander BRET sensors (27). Following validation of these assays (Fig. 5, *A*–*E*), we stimulated cells cotransfected with pcDNA, GPR35 short, or GPR35 long with one of the GPR35 agonists (Fig. 5, *F*–*T*, S7–S11 and Tables S1–S3). Here, ligand stimulation did not induce any GPR35-dependent responses in the readouts downstream of active G_i/o_, G_q/11_, or G_s_ (Fig. 5, *F*–*Q*). In contrast, translocation of PKN-RBD to the cell surface occurred specifically in cells expressing GPR35 isoforms (Fig. 5, *R*–*T*), confirming our results obtained with G_12/13_ biosensors in an assay system relying on endogenous expression levels of heterotrimeric G proteins. In contrast to the marginal increases in agonist potencies to induce G_13_ activation *via* GPR35 long, no statistical differences could be observed between the pEC_50_ values obtained with this assay. In further agreement, PKN-RBD recruitment was less pronounced upon agonist stimulation of GPR35 long, manifesting in a 40 to 50% reduction of the maximum BRET response compared to GPR35 short. Taken together, these data indicate that the extended N-terminus in GPR35 plays a negative allosteric modulatory role in receptor-mediated G protein activation.

### Ligand-induced GPR35 interaction with β-arrestins

**Figure 6:**
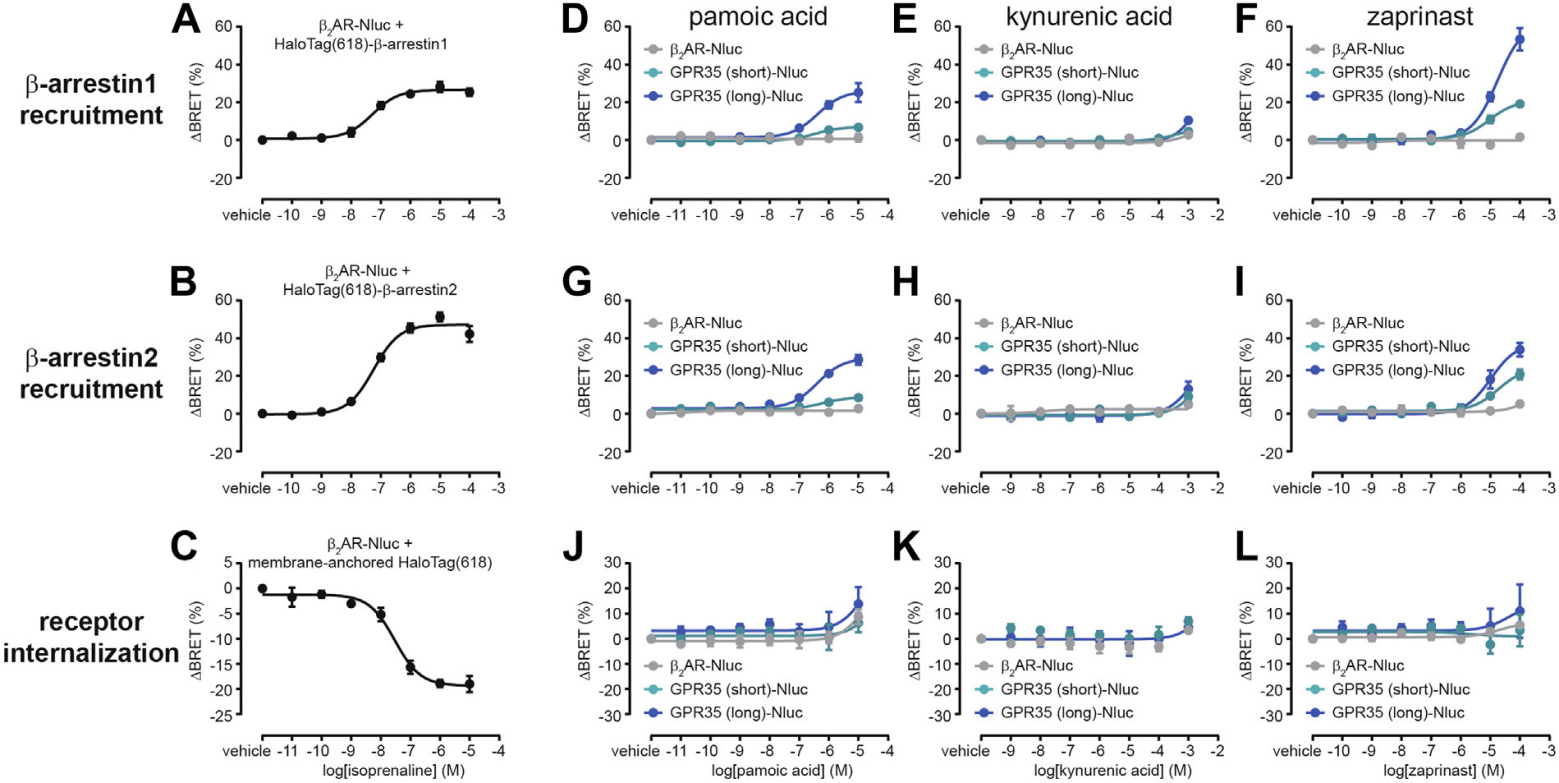
**GPR35–β-arrestin interaction and receptor internalization.***A* and *B*, recruitment of HaloTag-β-arrestin1 (*A*) or of HaloTag-β-arrestin2 (*B*) to isoprenaline-stimulated β_2_AR-Nluc. *C*, internalization of isoprenaline-stimulated β_2_AR-Nluc. *D*–*F*, recruitment of HaloTag-β-arrestin1 to pamoic acid- (*D*), kynurenic acid- (*E*), or zaprinast-stimulated (*F*) β_2_AR-, GPR35 short-, or GPR35 long-Nluc. *G*–*I*, recruitment of HaloTag-β-arrestin2 to pamoic acid- (*G*), kynurenic acid- (*H*), or zaprinast-stimulated (*I*) β_2_AR-, GPR35 short-, or GPR35 long-Nluc. *J*–*L*, internalization of pamoic acid- (*J*), kynurenic acid- (*K*), or zaprinast-stimulated (*L*) β_2_AR-, GPR35 short-, or GPR35 long-Nluc. Data show mean ± s.e.m. of three to four independent experiments conducted in transiently transfected HEK293A cells. Vehicle-treated ΔBRET values were set to 0. BRET, bioluminescence resonance energy transfer; HEK293, human embryonic kidney 293A; Nluc, NanoLuciferase.

Besides coupling to heterotrimeric G proteins, GPR35 isoforms interact with β-arrestin2, a process that depends on agonist-promoted phosphorylation of a species-conserved serine/threonine cluster in the receptor C-terminus (28). Following the profiling of G protein activation and signaling, we next investigated isoform-specific GPR35–β-arrestin interaction and receptor internalization. We employed an intermolecular BRET assay to monitor interaction of C-terminally Nluc-tagged GPR35 with fluorescently labeled HaloTag-β-arrestin1/2 (29) and assessed receptor translocation using a bystander BRET setup based on GPR35-Nluc and membrane-anchored, fluorescently labeled HaloTag (30, 31). All three assays were validated by activating the Nluc-tagged β_2_-adrenoreceptor (β_2_AR) with isoprenaline (Figs. 6, *A*–*C*, S12*A*, S13*A*, and S14*A*). In these assays, stimulating GPR35 short or long with the three agonists revealed that pamoic acid and zaprinast, but not kynurenic acid, promote β-arrestin1/2 recruitment to GPR35 isoforms (Figs. 6, *D*–*I*, S12, *B*–*J*, S13, *B*–*J*, Table S1–S3). In contrast to all G protein–dependent assays, pamoic acid and zaprinast promoted substantially enhanced BRET maxima through GPR35 long. The maximum BRET response induced by pamoic acid was more than 200% higher for GPR35 long *versus* short, indicating that the extended N-terminus plays a positive modulatory for arrestin recruitment. Intriguingly, no internalization of GPR35 could be observed following treatment with any of the three agonists (Figs. 6, *J*–*L* and S14, *B*–*J*).

### Allosteric control of constitutive and agonist-induced GPR35 activity through an extracellular disulfide bridge

**Figure 7:**
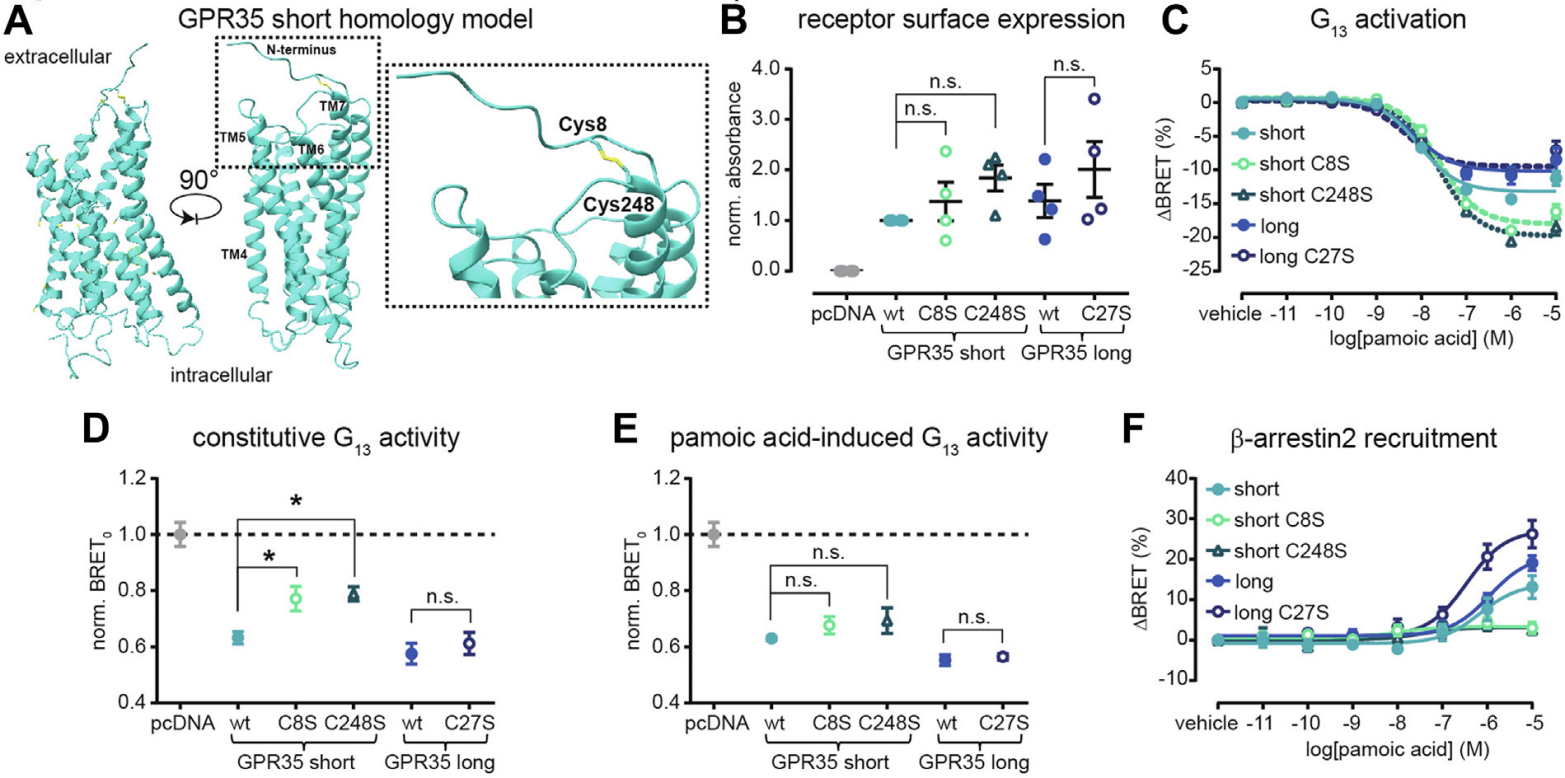
**Extracellular cysteine residues in GPR35 isoforms regulate constitutive and ligand-induced receptor-transducer coupling.***A*, homology model of GPR35 short indicating the formation of a disulfide bridge between Cys8 in the N-terminus and Cys248 in ECL3. *B*, surface expression levels of the indicated GPR35 isoform mutants and WT. The negative control (pcDNA transfection) was normalized to 0, and the expression of GPR35 short was set to 1.0 for each individual experiment. *C*, Pamoic acid–induced activation of the G_13_ activation mediated by the indicated GPR35 isoform mutants and WT. *D* and *E*, BRET_0_ analysis of G_13_ activity prior (*D*) and after (*E*) agonist-induced GPR35 activation. *F*, recruitment of β-arrestin2 to pamoic acid–stimulated GPR35 isoform mutants and WT. Data show mean ± s.e.m. of four to five independent experiments conducted in transiently transfected HEK293A cells. Statistical significance in (*B*), (*D*), and (*E*) was tested using one-way ANOVA followed by Tukey’s multiple comparison; *p* < 0.5. BRET, bioluminescence resonance energy transfer; HEK293, human embryonic kidney 293A.

The different patterns in G protein activation and β-arrestin recruitment between the GPR35 isoforms prompted us to elaborate on the structural basis underlying constitutive and ligand-induced activity of GPR35. A previous review on the therapeutic potential of GPR35 suggested that an additional cysteine residue located on the extended N-terminus of GPR35 long, Cys27, may cause differences in isoform function (32). Indeed, homology modeling of GPR35 short suggested—similar to structural predictions of AlphaFold2 (33) and in accordance with one of the templates (PDB 6RNK)—that a disulfide bridge is possible at the receptor’s extracellular surface formed by Cys8 of the (short) N-terminus and Cys248 in extracellular loop 3 (ECL3) (Figs. 7*A* and S15). In addition, we hypothesized that Cys27 of the long receptor isoform could affect GPR35 activity *in cellulo* by providing an additional, competing anchor for the formation of an alternative disulfide bond with either Cys8 or Cys248 (numbering referring to short isoform). To test this hypothesis, we generated three GPR35 point mutants, GPR35 long Cys27Ser (“long C27S”), GPR35 short Cys8Ser (“short C8S”), and GPR35 short Cys248Ser (“short C248S”) and assessed their activity in cell-based assays. Of note, none of the three point mutants showed altered surface expression levels compared to WT GPR35 isoforms (Fig. 7*B*). While the C27S mutation in GPR35 long did not affect GPR35-mediated G_13_ activation and only slightly abrogated downstream PKN-RBD recruitment, an increased agonist-mediated BRET change but slightly (about threefold) reduced pamoic acid potency was detected for the GPR35 short mutants Cys8Ser and Cys248Ser (Figs. 7*C*, S16, S17, and Table S4). Intriguingly, the amplitude of the change in GPR35 short mutants C8S compared to C248S was very similar. Comparison of the constitutive activity of these GPR35 mutants indicated that the increased ligand-induced BRET response detected with short C8S and short C248S could originate from their reduced capacity to activate G_13_ in absence of receptor agonists (Figs. 7*D* and S18*A*). In contrast, BRET_0_ analysis of the time points after agonist stimulation did not reveal statistically significant differences in G_13_ activity (Figs. 7*E* and S18*B*), suggesting that the ligand-stabilized, fully active state of GPR35 does not (or only marginally) depend on the proposed disulfide bridge between Cys8 and Cys248 (numbering refers to GPR35 short). In β-arrestin2 recruitment assays, we observed similarly intriguing effects of these mutations. While GPR35 long C27S elevated pamoic acid–induced receptor–arrestin interaction compared to WT, short C8S and short C248S showed substantially reduced capabilities to recruit β-arrestin2 (Figs. 7*F* and S19, *A*–*F*). Comparison of the baseline BRET values indicated that the altered extent in ligand-induced receptor-arrestin coupling is not due to distinct basal interactions of the mutants with β-arrestin2 (Fig. S19*G*). Additionally, we observed a three-fold increase in agonist potency for long C27S compared to WT GPR35 long (Table S4). Taken together, these data suggest that the proposed disulfide bridge between Cys8 and Cys248 (numbering refers to GPR35 short) and the additional cysteine residue in position 27 of GPR35 long are not involved in ligand–receptor interaction. However, our findings indicate that these structural elements differentially regulate constitutive and ligand-induced receptor interaction with G proteins (Cys8 and Cys248) and β-arrestins (all three Cys residues) through transmembrane allostery and thereby contribute to the observed transducer coupling bias of GPR35 isoforms.

## Discussion

The GPCRome represents one of the largest gene families in humans and is therefore involved in a variety of physiological processes. While biomedical research and drug discovery programs have focused on delineating and targeting the function of selected GPCRs for decades, alternative receptor isoforms originating from the same gene have long been neglected. The existence of a second GPR35 isoform has already been described in 2004 (5), but the genetic, regulatory mechanisms underlying isoform expression and a comprehensive pharmacological comparison of the signaling pathways promoted by GPR35 short and long are still pending. In the present study, we examined how alternative gene expression regulation leads to distinct isoform levels in immune and gastrointestinal cells and profiled GPR35 isoform–dependent cellular signaling in model HEK293 cells to investigate differences in isoform function.

Quantifying GPR35-mediated, constitutive activation of G proteins revealed that subtypes from three of the four major G protein families are activated in the presence of GPR35: G_z_ (G_i/o_ family), G_15_ (G_q/11_), G_12_ and G_13_ (both G_12/13_). While constitutive activation of G_15_ and G_12_ has, to the best of our knowledge, not been reported so far, ligand-independent activation of G_13_ is in line with earlier studies that utilized a chimeric G_q_/G_12/13_ protein (34). Furthermore, ligand-independent activation of G_z_ is supported by the previous observation that GPR35 overexpression suppresses forskolin-induced elevation of intracellular cAMP levels (35). Treatment with three different receptor agonist further revealed that GPR35 preferentially activates and signals *via* G_12/13_ proteins. Surprisingly, constitutive activation of G_z_ was not further elevated upon agonist stimulation and the activity of G_15_ was increased only at the highest ligand concentrations.

We further observed several intriguing similarities and differences in GPR35-mediated intracellular signaling compared to previous studies. For instance, GPR35 inhibited forskolin-induced cAMP production in a pertussis toxin–sensitive manner in nociceptive neurons of rat dorsal root ganglia (36). Our own data from HEK293 cells, however, did not reveal GPR35-dependent alterations in intracellular cAMP levels despite modest activation of G_o1_ at highest zaprinast concentrations (this work) and of chimeric G_o/q_ in Chinese hamster ovarian cells (21). Likewise, our previous work in primary adipocytes showed that kynurenic acid–mediated activation of GPR35 promotes intracellular Ca^2+^ signaling, which was attributed to GPR35-G_i/o_ coupling (7). While our present work does, however, not provide convincing evidence for GPR35-G_i/o_ or GPR35-G_q_ signaling in HEK293 cells, the constitutive elevation of NFAT-RE reporter gene activity upon GPR35 coexpression in these cells indicates that Ca^2+^ plays yet a central role in GPR35-mediated signaling. Furthermore, fluorescence imaging experiments showed ligand-induced internalization of GPR35 in cardiomyocytes and U2OS osteosarcoma cells (16, 24), but our validated BRET setup did not detect agonist-induced clearance of GPR35. Taken together, these differences in receptor-transducer coupling, second messenger elevations and receptor internalization suggest that the function of GPR35 highly depends on the cellular context and/or its expression levels in the different test systems.

Comparing the two GPR35 isoforms in cellular signaling experiments led to additional compelling observations. As already shown previously with readouts of Gα13 and β-arrestin2 coupling to GPR35 (1), the extended N-terminus of GPR35 long did not—or only slightly in the case of G_13_—affect the potency of any of the three GPR35 agonists, indicating that the N-terminal extension by 31 amino acids does not contribute to or allosterically modulate the shape of the receptor’s ligand-binding pocket(s). However, the maximum BRET responses were affected in a consistent pattern: GPR35 short provoked higher BRET amplitudes in all G protein–dependent assays, while GPR35 long evoked higher BRET amplitudes in β-arrestin recruitment assays, demonstrating that the extended N-terminus allosterically modulates the active agonist-bound receptor conformation. Allosteric modulation by the extended N-terminus results in altered efficacy of the receptor to couple to and activate its intracellular transducer proteins and therefore confers bias toward β-arrestin. Recently, N-terminal regulation of GPCR pathway bias toward β-arrestins was also reported for a shorter splice variant of the growth hormone–releasing hormone receptor, presumably because the alternative N-terminus alters the intracellular receptor-arrestin interface (4). Transmembrane allosteric control of receptor-transducer coupling by extracellular receptor domains has also been described for β_2_AR. Here, a single phenylalanine residue in extracellular loop 2 is crucial for receptor–β-arrestin2 interaction following agonist binding (37). In contrast to these two studies, coupling efficacy was altered for GPR35, underlining that different GPCRs have evolved distinct mechanisms to modify receptor function through transmembrane allostery.

To gain information about the underlying structural mechanisms causing the observed pathway bias of GPR35 isoforms, we combined receptor homology modeling and mutagenesis studies *in-vitro*. Our experiments revealed that a proposed disulfide bridge connecting the receptor’s N-terminus with ECL3 contributes to constitutive activation of G_13_ by GPR35 and agonist-induced recruitment of β-arrestin2. In contrast, a cysteine residue provided by the extended N-terminus of GPR35 long (Cys27) has no effect on (constitutive or ligand-mediated) G_13_ activation but abrogates receptor-arrestin coupling by reducing agonist potency and efficacy. How this effect is accomplished remains, however, unclear. Possible explanations could involve competition for disulfide bridge formation within the same GPR35 protomer (*e.g.*, with Cys8 or Cys248; numbering referring to GPR35 short) or even across GPR35 molecules in receptor dimers that are suggested to be formed in corneal tissues (38). Conclusively, these structural insights provided by the experiments with the GPR35 Cys-to-Ser mutants cannot entirely explain the molecular mechanism by which the extended N-terminus of GPR35 long alters receptor function, yet they could aid in designing potent and efficacious ligands that regulate GPR35 coupling to G proteins and arrestins with transducer selectivity.

Taken together, our study provides insights into the complex mechanism of transmembrane allostery conformational regulation of GPCR isoform function and provides clues for strategies to target-biased GPCR signaling *in vivo*. For instance, selective GPR35 ligands could be functionally derivatized in order to chemically modify functionally relevant cysteine residues—as has been done previously to covalently label endogenous GPCRs (39)—and regulate GPR35 activity in an isoform-specific fashion.

## Experimental procedures

### Plasmids and molecular cloning

The expression plasmid encoding N-terminally HA-tagged GPR35 short isoform was obtained from the cDNA resource center (cdna.org, cat. no. GPR035TN00). The insert was amplified by PCR with NotI and XhoI overhangs and inserted into the pcDNA3-Flag expression plasmid. For GPR35 long, a G-block dsDNA (from IDT) corresponding to the sequence between the NotI and HaeII restriction sites with the N-terminally added sequence encoding the additional 31 aa was inserted into the pcDN3-Flag expression plasmid. The sources for plasmids encoding thromboxane A_2_ receptor, histamine H_3_ receptor, membrane-anchored HaloTag BRET sensors for G_i1_, G_i2_, G_i3_, G_o1_, G_s_, G_q_, G_15_, and G_12_ (tricistronic G protein activity sensors; available from Addgene), the FRET sensor H187, CRE-Fluc, SRE-Fluc, NFAT-RE-Fluc SRF-RE-Fluc were described previously (25, 30, 40). WT H_2_R was purchased from cDNA.org in pcDNA and biosensor components to assess p63RhoGEF and PKN-RBD recruitment and DAG generation were kindly provided by Prof. Michel Bouvier (Université de Montréal) under a material transfer agreement. Plasmids encoding HaloTag-labeled β-arrestin1 and 2 were kindly provided by Prof. Carsten Hoffmann (Friedrich-Schiller-University Jena). The sensors for G_12_ and G_z_ as well as C-terminally Nluc-tagged GPR35 isoforms and β_2_AR were generated using established PCR and restriction enzyme strategies. The G_12_ sensor is composed of Gβ3-T2A-cpVenus-Gγ9 and Nluc-tagged Gα12 (Nluc inserted with a flexible SG-linker between amino acids #133 and #134 of Gα12). The sensor for G_z_ is composed of pGβ1-T2A-cpVenus-Gγ1 and Nluc-tagged Gαz (Nluc inserted with a flexible SG-linker between amino acids #113 and #114 of Gαz). Site-directed mutagenesis was performed using PrimeSTAR Max DNA Polymerase (Takara) PCR with mutation containing primers and subsequent DpnI digestion to eliminate template DNA. All constructs were verified by sequencing (Eurofins genomics).

### Reagents

Imetit, histamine, poly-D-lysine, isoprenaline, and U-46619 were obtained from Sigma Aldrich (Merck KGaA). Zaprinast, kynurenic acid, and pamoic acid were from Sigma-Aldrich, Tocris Bioscience and Sigma-Aldrich, respectively. The Nluc substrate furimazine and the HaloTag fluorescent ligand NanoBRET 618 were from Promega (Madison). Coelenterazine 400a was from Biosynth. White-wall, white-bottomed 96-well, black-wall, black bottomed 96-well, as well as flat-bottomed transparent microtiter plates were from Gibco.

### Splicing analysis

Data for small intestine and monocytes were downloaded from ENCODE (experiments: ENCSR905LVO, ENCSR000CUC, ENCSR719HRO, ENCSR612HYR, ENCSR618IQY) as fastq files. These were aligned to the human genome (GRCm38) using STAR (version 2.7.3a) (41) with the following parameters “--twopassMode Basic --outFilterMismatchNmax 10 --outFilterScoreMinOverLread 0.7 --outFilterMatchNminOverLread 0.7 --outSAMstrandField intronMotif --outFilterMultimapNmax 50 --outFilterMultimapScoreRange 3 --alignIntronMax 500000 --alignMatesGapMax 1000000 --sjdbScore 2done”. Resulting bam files were plotted as sashimi plots using the ggsashimi tool (42). Only junctions supported by more than 30 reads on average are shown.

### Cell culture

HEK293A cells were used for transient expression of the indicated GPCRs and biosensors and grown in Dulbecco’s Modified Eagle’s Medium (DMEM) supplemented with 2 mM glutamine, 10% fetal calf serum, 0.1 mg/ml streptomycin, and 100 units/ml penicillin at 37 °C with 5% CO_2_. Absence of *mycoplasma* contamination was routinely confirmed by PCR.

### Transient transfection and plating

Resuspended cells (300,000 cells/ml) were transfected in suspension with a total of 1 μg DNA/ml suspension using Lipofectamine 2000 (Thermo Fisher Scientific; 2 μl Lipofectamine 2000 per μg DNA). For reporter gene experiments, resuspended cells were transfected with 500 ng pcDNA or the indicated receptor along with 100 ng pRL-TK Luc (expression control) and 400 ng of the indicated reporter gene-Fluc plasmid per ml cell suspension. For transfection of GPR35 isoforms only for surface expression quantification, 500 ng of the plasmids was mixed with 500 ng pcDNA per ml cell suspension. For cotransfection of GPCRs along with the tricistronic G protein, the cAMP FRET or the DAG sensor, 500 ng GPR35 or pcDNA and 500 ng sensor plasmid were combined. For PKN-RBD and p63RhoGEF recruitment assays, 500 ng GPR35 or pcDNA was combined with 100 ng rGFP-CAAX and 400 ng RlucII-tagged PKN-RBD or p63RhoGEF. For internalization experiments, 100 ng GPCR-Nluc was combined with 400 ng membrane-anchored HaloTag and 500 ng pcDNA. To measure β-arrestin recruitment Nluc-tagged GPCRs, 333 ng GPCR-Nluc was combined with 667 ng of HaloTag-labeled β-arrestin. To measure β-arrestin2 recruitment to unlabeled GPR35 constructs, 500 ng receptor or pcDNA were combined with 400 ng of membrane-anchored HaloTag and 100 ng Nluc-β-arrestin2 per ml cell suspension. Cells mixed with the transfection reagents were seeded onto poly-D-lysine–precoated 96-well plates and grown for 48 h at 37 °C with 5% CO_2_. White plates were used for BRET and reporter gene experiments, black plates for cAMP FRET experiments and transparent, and flat bottom 96-well plates were used for the assessment of GPR35 surface levels.

### Assessment of GPR35 receptor surface expression through live-cell ELISA

For quantification of cell surface receptor expression, HEK293A cells transfected with pcDNA or N-terminally FLAG-tagged GPR35 isoforms were grown for 48 h in transparent 96-well plates and washed once with 0.5% bovine serum albumin (BSA) in PBS. Next, cells were incubated with a rabbit anti-FLAG-tag antibody (1 μg/ml, cat# F7425; Sigma Aldrich) in 1% BSA–PBS for 1 h at 4 °C. Following incubation, the cells were washed three times with 0.5% BSA–PBS and incubated with a horseradish peroxidase–conjugated goat anti-rabbit antibody (0.3 μg/ml, cat# 31460; Thermo Fisher Scientific) in 1% BSA–PBS for 1 h at 4 °C. The cells were washed three times with 0.5% BSA/PBS, and 50 μl of the peroxidase substrate 3,3′,5,5′-tetramethylbenzidine (T8665; Sigma-Aldrich) was added. Subsequently, the cells were incubated for 20 min and 50 μl of 2 M HCl was added. The absorbance was read at 450 nm using a BMG Ω POLARstar plate reader.

### Ligand-induced FRET and BRET measurements

Transfected cells grown for 48 h in 96-well plates were washed with Hank's balanced salt solution (HBSS) and incubated with 1/1000 dilution of furimazine stock solution (for Nluc-based sensors) or with 5 μM coelenterazine 400a. No substrate was added to HBSS for FRET-based cAMP experiments. After incubation for 3 min at 37 °C, the FRET and BRET ratio was measured in at least three consecutive reads followed by addition of ligand solutions or vehicle control and subsequent FRET/BRET reads to detect ligand-induced changes in energy transfer. All experiments were conducted at 37 °C. Nluc emission intensity was selected using a 450/40 nm monochromator and cpVenus emission using a 535/30 nm monochromator or HaloTag NanoBRET 618 emission using a 630/60 nm monochromator in a CLARIOstar plate reader with an integration time of 0.3 s. Renilla luciferase (RlucII) emission intensity was selected using a 400/80 nm monochromator and rGFP emission using a 525/70 nm monochromator using a Tecan Spark plate reader with an integration time of 0.1 s. FRET was measured using a CFP/YFP filter pair (excitation: 430/20 nm; emission 480/20 and 535/20 nm) using the CLARIOstar plate reader with 40 flashes per data point.

### BRET_0_-based assessment of constitutive GPCR activity

Transfected cells grown for 48 h in 96-well plates were washed with HBSS and incubated with 1/1000 dilution of furimazine stock solution. After incubation for 3 min at 37 °C, the BRET ratio was measured for the assessment of constitutive receptor activity. All experiments were conducted at 37 °C with a CLARIOstar plate reader. Nluc emission intensity was selected using a 450/40 nm monochromator and cpVenus emission using a 535/30 nm monochromator with an integration time of 0.3 s in both channels.

### Reporter gene activity assays

Transfected cells grown in 96-well plates were washed with 100 μl HBSS 24 h posttransfection and incubated for another 24 h in FBS-reduced (0.5%) DMEM. The day of the experiment, cells were washed with HBSS and lysed in 30 μl of Promega’s dual luciferase passive lysis buffer (15 min, room temperature). Next, 20 μl luciferase assay reagent was added to each well and reporter gene activity–dependent firefly luciferase intensity was measured using a Tecan Spark microplate reader (585/70 nm; 1 s integration time). Next, 20 μl Stop&Glo Reagent was added to quantify Rluc emission intensity (487/85 nm; 1 s integration time) to control for variations in cell number and transfection efficiency.

### Data analysis

Reporter gene activity was expressed as firefly luciferase over Rluc luminescence intensity. FRET and BRET ratios were defined as acceptor emission/donor emission. The basal FRET/BRET ratio before ligand stimulation (Ratio_basal_) was defined as the average of at least three consecutive reads. To quantify ligand-induced changes, ΔFRET/BRET was calculated for each well as a percent over basal [(Ratio_stim_−Ratio_basal_)/Ratio_basal_] × 100). Subsequently, the average ΔFRET/BRET of vehicle control was subtracted. Data were analyzed using Prism 5.0 software (GraphPad). Data from FRET and BRET concentration–response experiments were fitted using a four-parameter fit. Nluc/BRET plots were fitted using a linear fit and tested for deviation from linear correlation applying runs test (*p* < 0.05). BRET_0_ was defined as the Y-intercept with its computed standard error resulting from the linear fit of BRET values over increasing donor emission intensities. Differences between BRET_0_ values were tested for significance using one-way ANOVA followed by Dunnett’s or Tukey’s multiple comparison as indicated in the figure legends. Data from cell surface ELISA experiments were corrected for background by subtracting the values obtained for pcDNA-transfected cells and normalized to GPR35 short WT. Selected differences in surface expression were tested for significance using one-way ANOVA followed by Tukey’s multiple comparison. *p* values < 0.05 were considered significant. Differences in agonist potency or efficacy were assessed by first determining whether the acquired concentration response curves were statistically different from negative control (pcDNA or β_2_AR-Nluc) to exclude GPR35-unrelated effects. Therefore, extra–sum-of-squares F-test were performed in order to determine whether one fit would represent all samples. If this pretest indicated different fits to represent the datasets, pEC_50_ and maximum ΔFRET/BRET values were compared between GPR35 short and long or between the Cys-to-Ser mutants and the respective WT isoform. Statistical differences were indicated in Tables S1–S4.

### Homology modeling of GPR35 short

The homology model of GPR35 short was created with Modeller10.2 based on multiple template alignment. Experimental structures of the lysophosphatidic acid receptor 6 (PDB 5XSZ), the succinate receptor (PDB 6RNK), the CC chemokine receptor 5 (PDB 5UIW), and the cysteinyl leukotriene receptors 1 (PDB 6RZ5) and 2 (PDB 6RZ6) were selected as templates for different regions of GPR35 following receptor similarity search to GPR35 in GPCRdb (43). The generated GPR35 model was visually inspected and superimposed with the AlphaFold2-predicted structure of GPR35 (33) to control for major deviations in the receptor’s tertiary organization. All models were visualized, and distances between selected residues were measured using the molecular visualization program UCSF ChimeraX (44) with the aim to exclude possible clashes, helical breaks, and high energy kinks in the predicted structure. Only distances below 2.1 Å between appropriately oriented sulfur atoms were considered for the formation of potential disulfide bridges.

The receptor sequence alignment of GPR35 short and long presented in Figure 2 was created using the EMBOSS Needle online tool (https://www.ebi.ac.uk/Tools/psa/emboss_needle/) (45).

## Data availability

All data are contained within the article.

## Supporting information

This article contains supporting information.

## Conflict of interest

The authors declare that they have no conflicts of interest with the contents of this article.
