# Supplementary material for: Isoforms of GPR35 have distinct extracellular N-termini that allosterically modify receptor-transducer coupling and mediate intracellular pathway bias
Source: J Biol Chem. 2022 Aug 4;298(9):102328. doi: 10.1016/j.jbc.2022.102328 (PMC9450150; doi:10.1016/j.jbc.2022.102328)
Supplement: Supporting information [file mmc1.docx]

**Title**

Isoforms of GPR35 have distinct extracellular N-termini that allosterically modify receptor-transducer coupling and mediate intracellular pathway bias

**Authors**

Hannes Schihada^1,2^*, Thomas M. Klompstra^1^, Laura J. Humphrys^3^, Igor Cervenka^1^, Shamim Dadvar^1^, Peter Kolb^2^, Jorge L. Ruas^1^, Gunnar Schulte^1^*

**Affiliations**

^1^: Department of Physiology and Pharmacology, Karolinska Institutet, 171 77, Stockholm, Sweden.
^2^: Department of Pharmaceutical Chemistry, Philipps-University Marburg, 35037 Marburg, Germany.
^3^: Institute of Pharmacy, University of Regensburg, 93053 Regensburg, Germany.

**Supporting Information**

fig. S1: Spaghetti plot of the surface expression data shown in Fig. 2.

fig. S2: Validation of G_z_ and G_12_ BRET sensors.

fig. S3: BRET over Nluc plots for assessment of constitutive GPR35 activity.

fig. S4: G protein BRET sensor time courses upon stimulation with pamoic acid.

fig. S5: G protein BRET sensor time courses upon stimulation with kynurenic acid.

fig. S6: G protein BRET sensor time courses upon stimulation with zaprinast.

fig. S7: cAMP FRET sensor time courses following forskolin pre-incubation.

fig. S8: p63RhoGEF recruitment time courses.

fig. S9: DAG generation time courses.

fig. S10: cAMP FRET sensor time courses.

fig. S11: PKN-RBD recruitment time courses.

fig. S12: β-arrestin1 recruitment time courses.

fig. S13: β-arrestin2 recruitment time courses.

fig. S14: Receptor internalization time courses.

fig. S15: Sequence alignment of GPR35 short to multiple templates for model building.

fig. S16: G_13_ BRET sensor time courses of GPR35 mutants.

fig. S17: PKN-RBD recruitment time courses and concentration response curves for GPR35 mutants.

fig. S18: BRET over Nluc plots for assessment of constitutive G_13_ activation by GPR35 mutants.

fig. S19: β-arrestin2 recruitment time courses and basal BRET values for GPR35 mutants.

table S1: EC_50_ values of pamoic acid and maximum ΔFRET/BRET responses at vector-, GPR35 short- or GPR35 long-transfected cells determined with different pharmacological assays.

table S2: EC_50_ values of kynurenic acid and maximum ΔFRET/BRET responses at vector-, GPR35 short- or GPR35 long-transfected cells determined with different pharmacological assays.

table S3: EC_50_ values of zaprinast and maximum ΔFRET/BRET responses at vector-, GPR35 short- or GPR35 long-transfected cells determined with different pharmacological assays. table S4: EC_50_ values of pamoic acid at GPR35 point mutants.

table S4: EC_50_ values and maximum ΔFRET/BRET responses of pamoic acid at GPR35 point mutants.


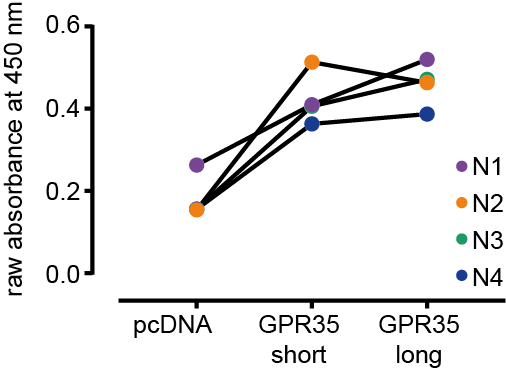


**Figure S1: Spaghetti plot of the surface expression data shown in Fig. 2.** Presented are the raw absorbance values of four independent experiments that were corrected for background (pcDNA) and normalized to GPR35 short to obtain the graph shown in Fig. 2.


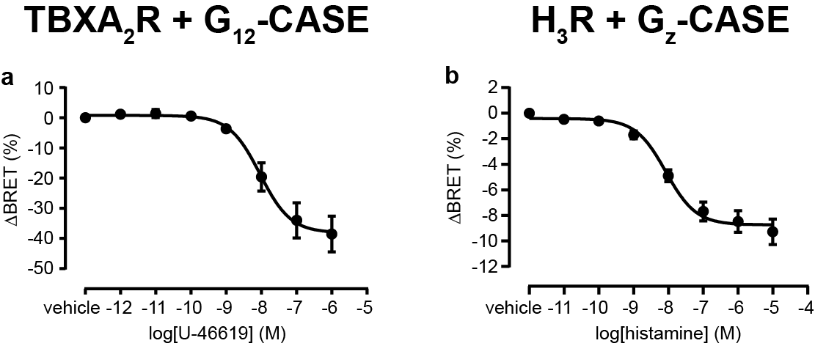


**Figure S2: Validation of G_z_ and G_12_ BRET sensors. a)** ΔBRET time course of U-46619-mediated activation of the thromboxane A_2_ receptor (TBXA_2_R) and subsequent G_12_ activation. **b)** ΔBRET time course of histamine-mediated activation of the histamine H_3_ receptor (H_3_R) and subsequent G_z_ activation. All experiments were conducted in HEK293A (a) or HEK293T cells co-transfected with the indicated GPCR/G protein sensor combination. Data represents mean ± SEM of three to four independent experiments.


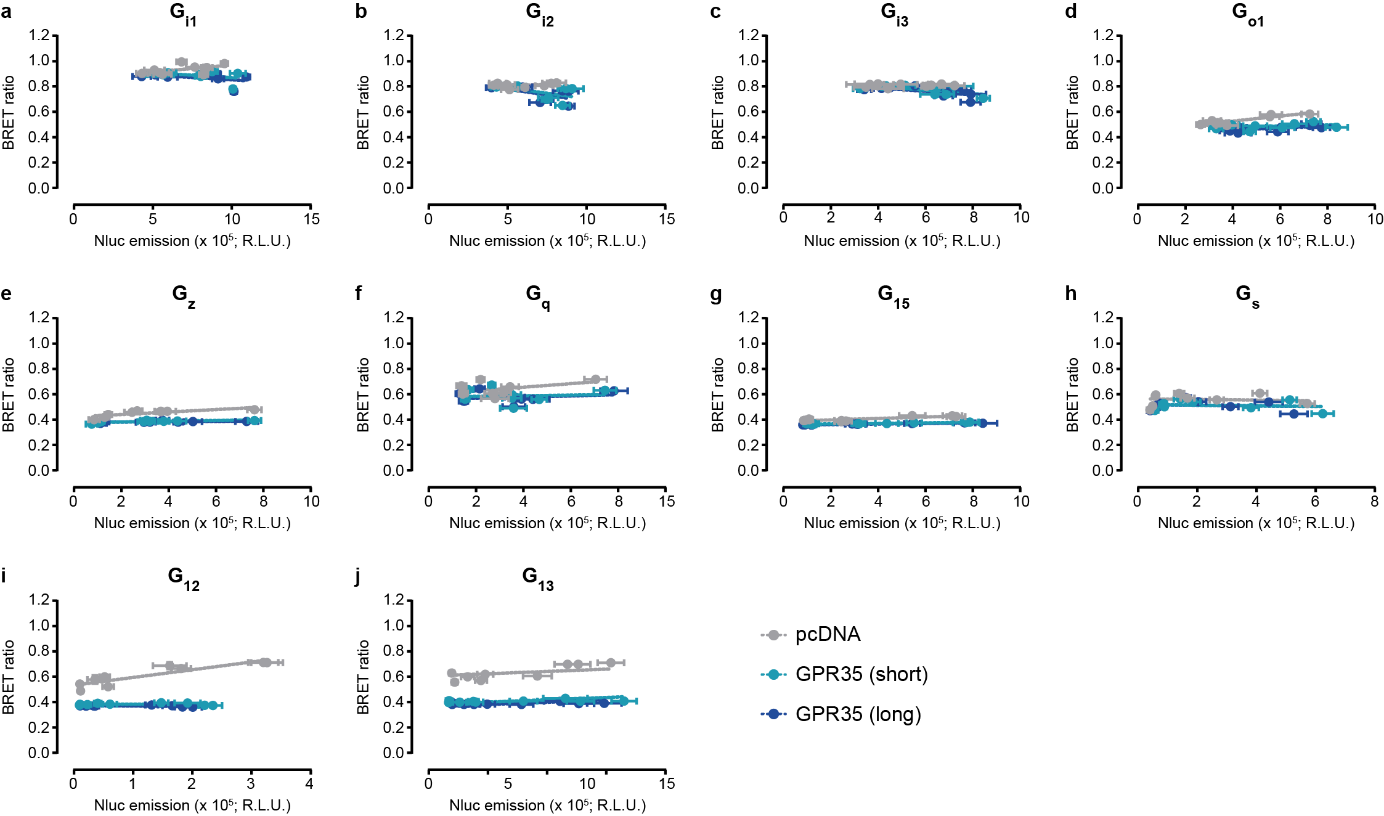


**Figure S3: BRET over Nluc plots for assessment of constitutive GPR35 activity.** Data points were fitted to a linear regression curve to determine BRET_0_ values presented in Fig. 2. All experiments were conducted in HEK293A cells co-transfected with the indicated GPCR or pcDNA/G protein sensor combination. Data represents mean ± SD of nine independent experiments.

**
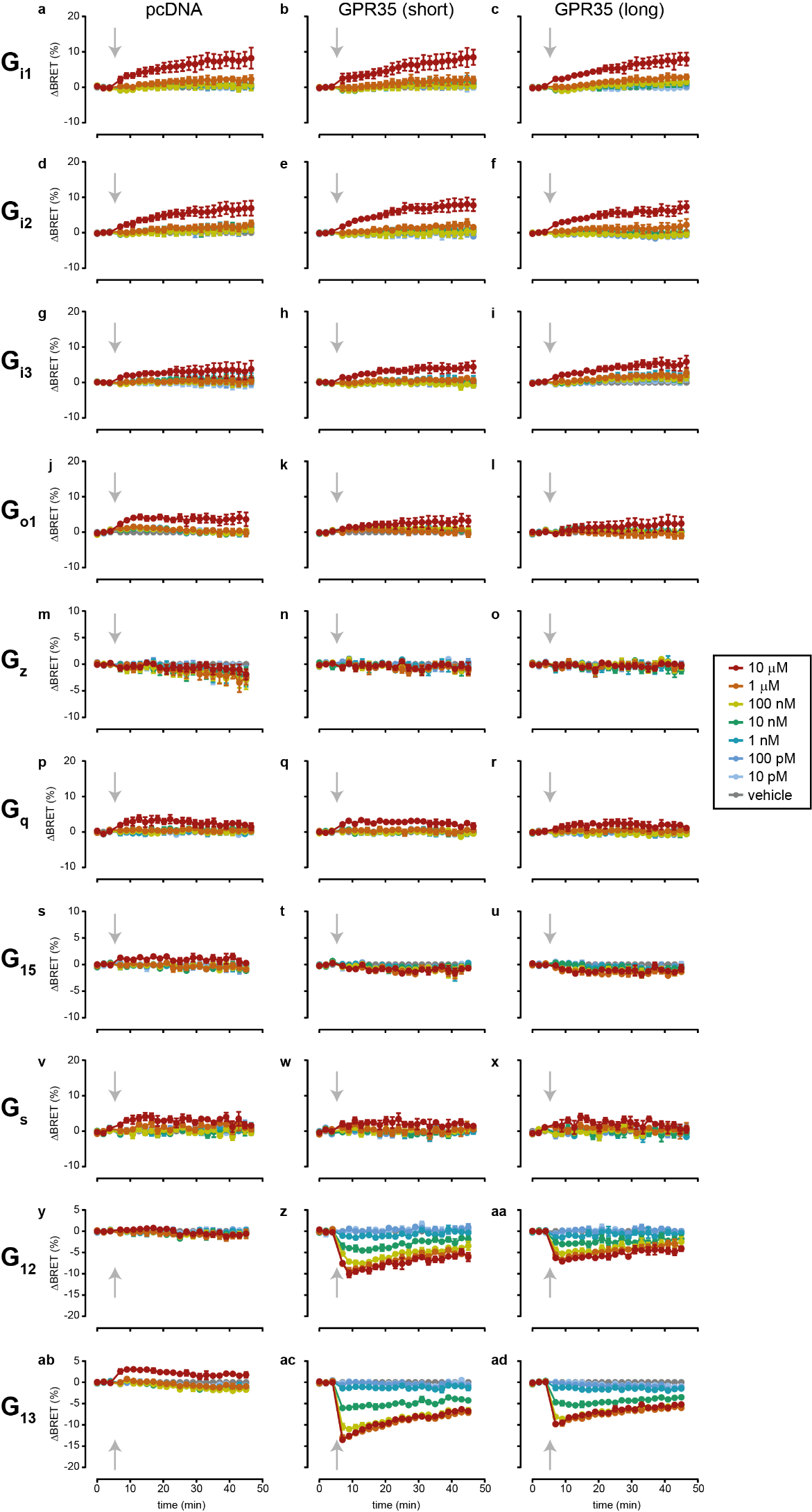
**

**Figure S4: G protein BRET sensor time courses upon stimulation with pamoic acid.** The grey arrow indicates the time point ligand or vehicle addition. All experiments were conducted in HEK293A cells co-transfected with the indicated GPCR or pcDNA/G protein sensor combination. Data represents mean ± SEM of three to four independent experiments. The grey arrow indicates the time point ligand or vehicle addition.

**
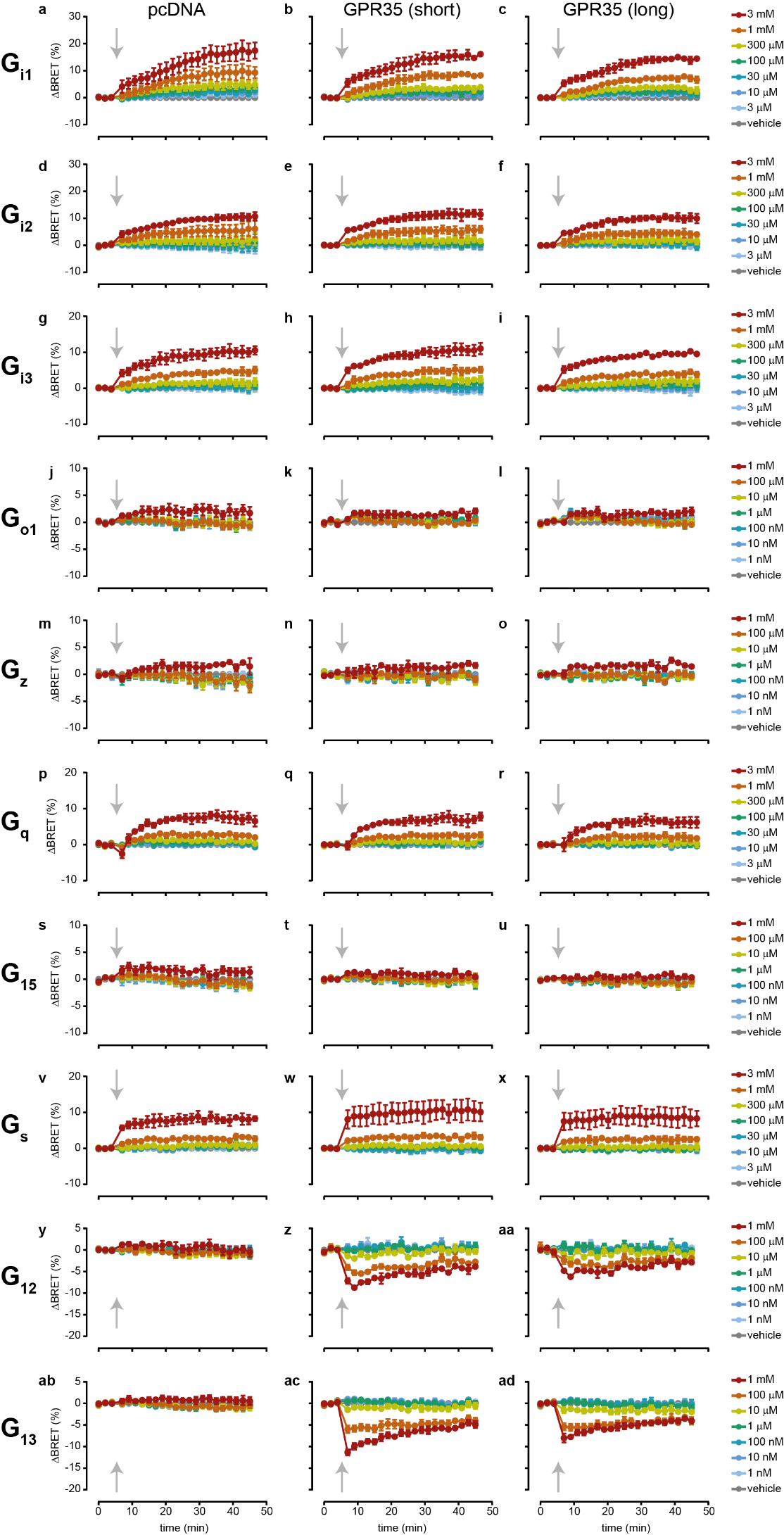
**

**Figure S5: G protein BRET sensor time courses upon stimulation with kynurenic acid.** The grey arrow indicates the time point ligand or vehicle addition. All experiments were conducted in HEK293A cells co-transfected with the indicated GPCR or pcDNA/G protein sensor combination. Data represents mean ± SEM of three to four independent experiments. The grey arrow indicates the time point ligand or vehicle addition.


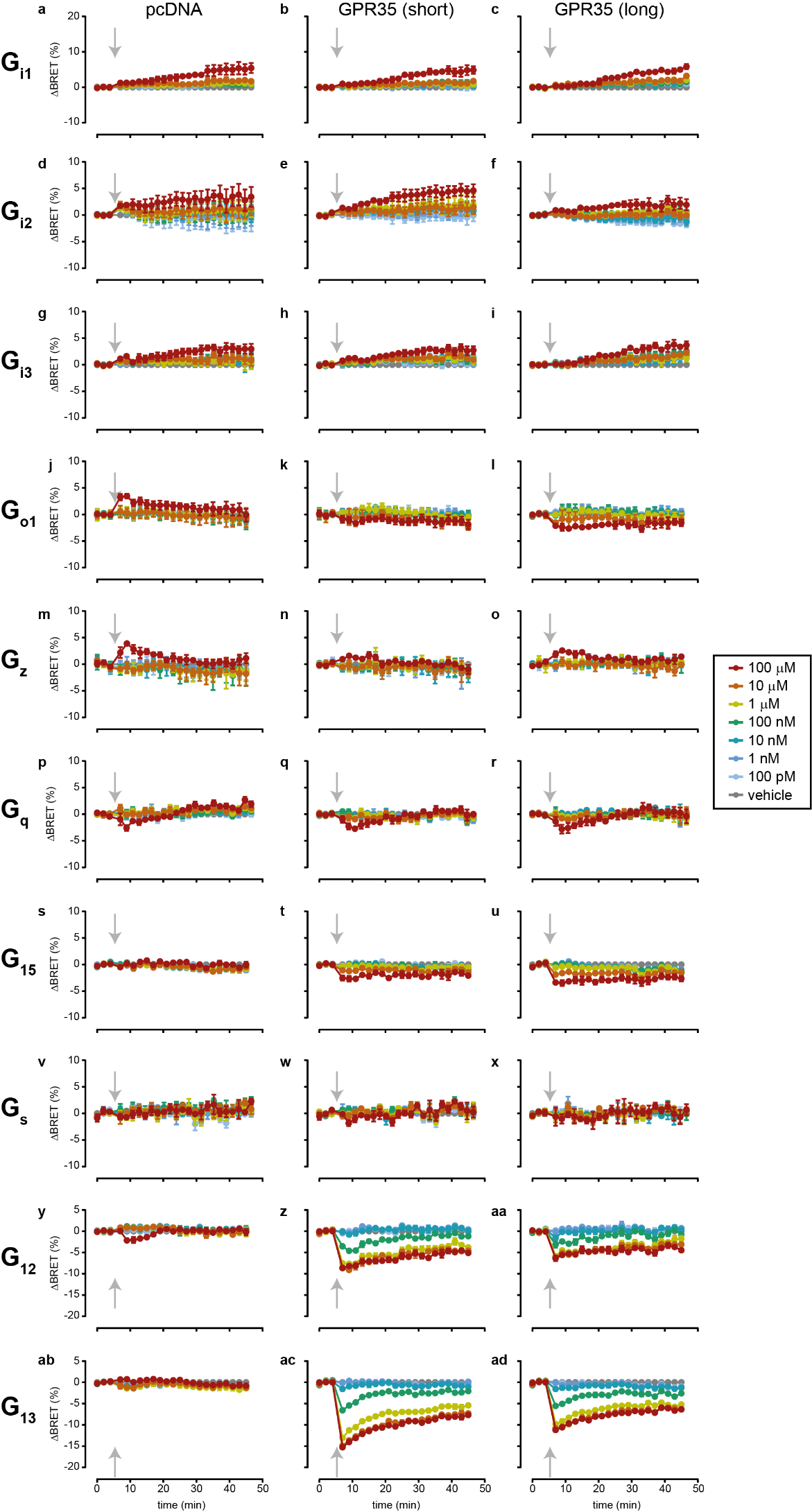


**Figure S6: G protein BRET sensor time courses upon stimulation with zaprinast.** The grey arrow indicates the time point ligand or vehicle addition. All experiments were conducted in HEK293A cells co-transfected with the indicated GPCR or pcDNA/G protein sensor combination. Data represents mean ± SEM of three to four independent experiments. The grey arrow indicates the time point ligand or vehicle addition.


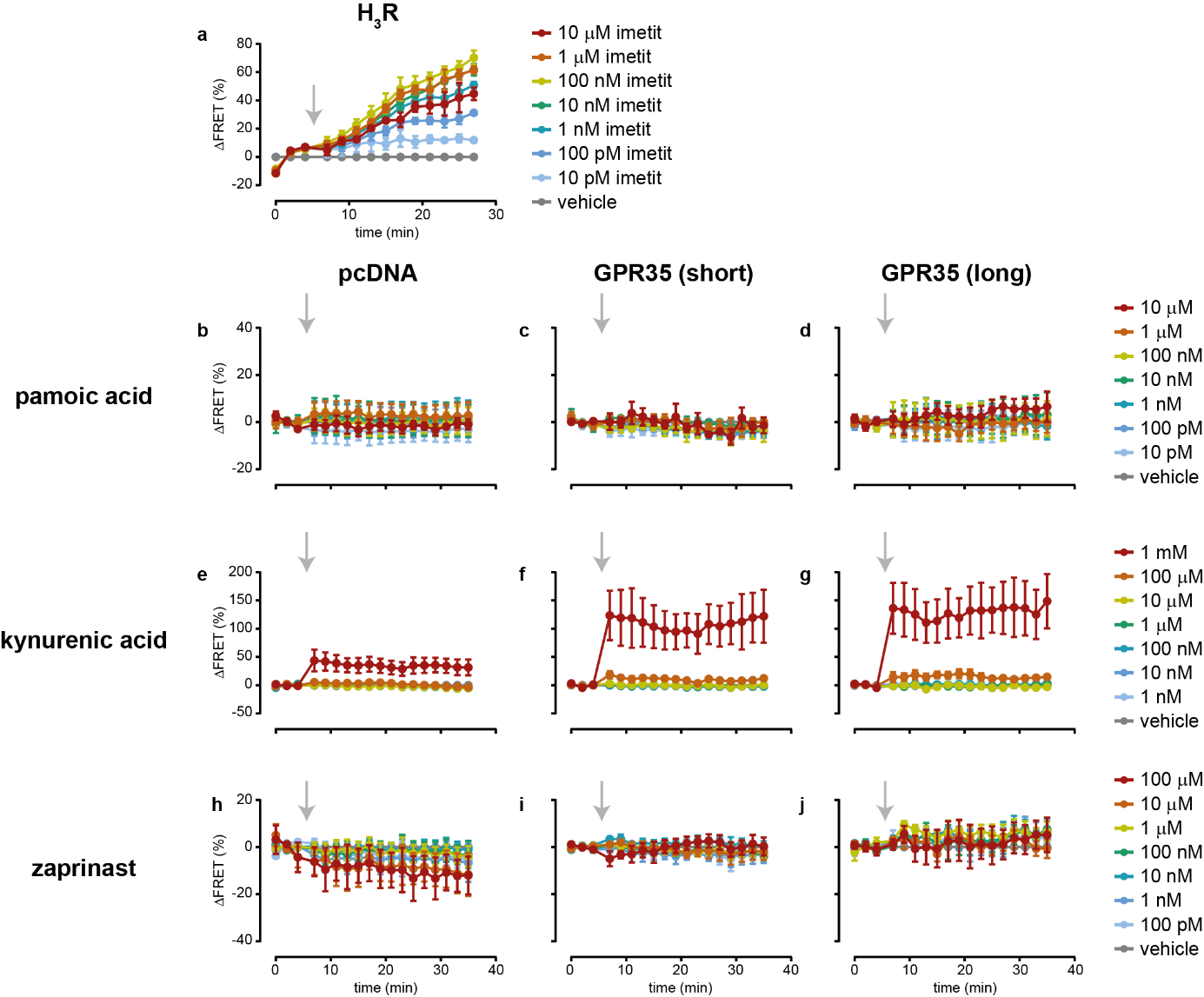


**Figure S7: cAMP FRET sensor time courses following forskolin pre-incubation. a)** Histamine H_3_ receptor (H_3_R)-mediated increases in FRET following stimulation with imetit. **b-j)** FRET changes induced by pamoic acid (b-d), kynurenic acid (e-g) or zaprinast (h-j) in cells co-transfected with the cAMP FRET sensor and either pcDNA or GPR35 isoforms. All experiments were conducted in HEK293A cells pre-incubated with 5 μM forskolin for five minutes. Data represents mean ± SEM of three independent experiments. The grey arrow indicates the time point ligand or vehicle addition.

**
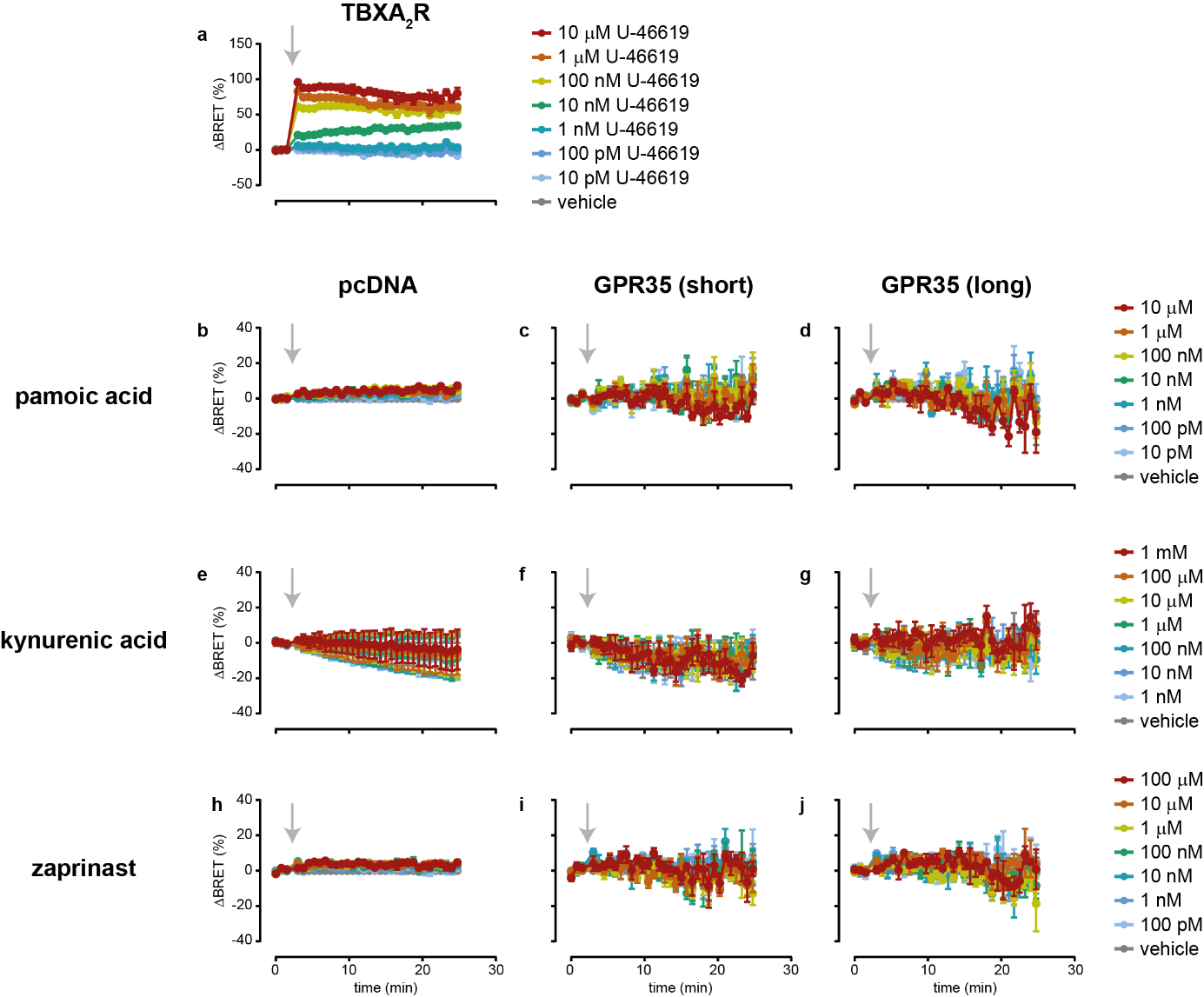
**

**Figure S8: p63RhoGEF recruitment time courses. a)** Thromboxane A_2_ receptor (TBXA_2_R)-mediated increases in BRET following stimulation with U-46619. **b-j)** BRET changes induced by pamoic acid (b-d), kynurenic acid (e-g) or zaprinast (h-j) in cells co-transfected with the p63RhoGEF recruitment sensor and either pcDNA or GPR35 isoforms. All experiments were conducted in HEK293A cells. Data represents mean ± SEM of three independent experiments. The grey arrow indicates the time point ligand or vehicle addition.


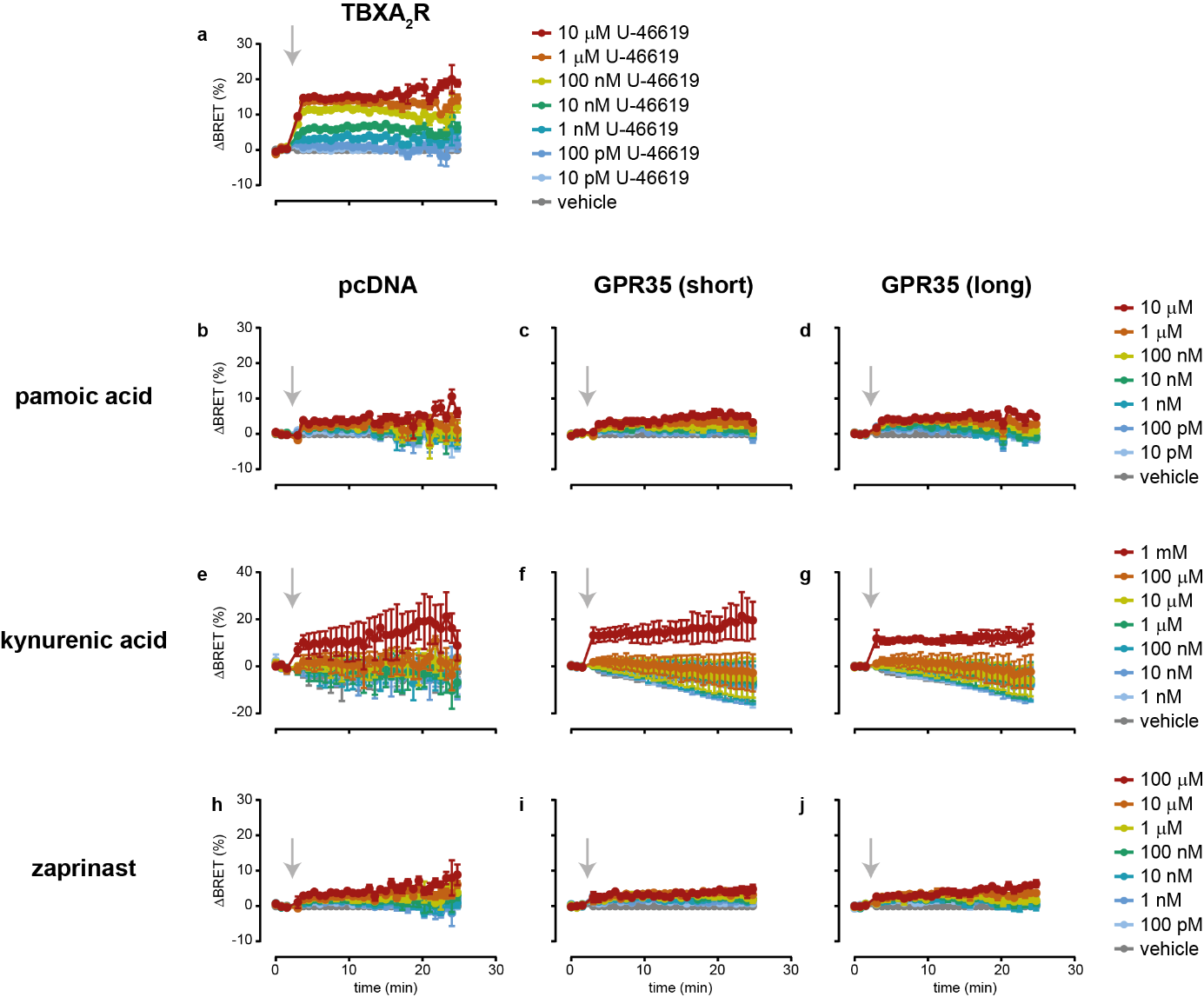


**Figure S9: DAG generation time courses. a)** Thromboxane A_2_ receptor (TBXA_2_R)-mediated increases in BRET following stimulation with U-46619. **b-j)** BRET changes induced by pamoic acid (b-d), kynurenic acid (e-g) or zaprinast (h-j) in cells co-transfected with the DAG BRET sensor and either pcDNA or GPR35 isoforms. All experiments were conducted in HEK293A cells. Data represents mean ± SEM of three independent experiments. The grey arrow indicates the time point ligand or vehicle addition.


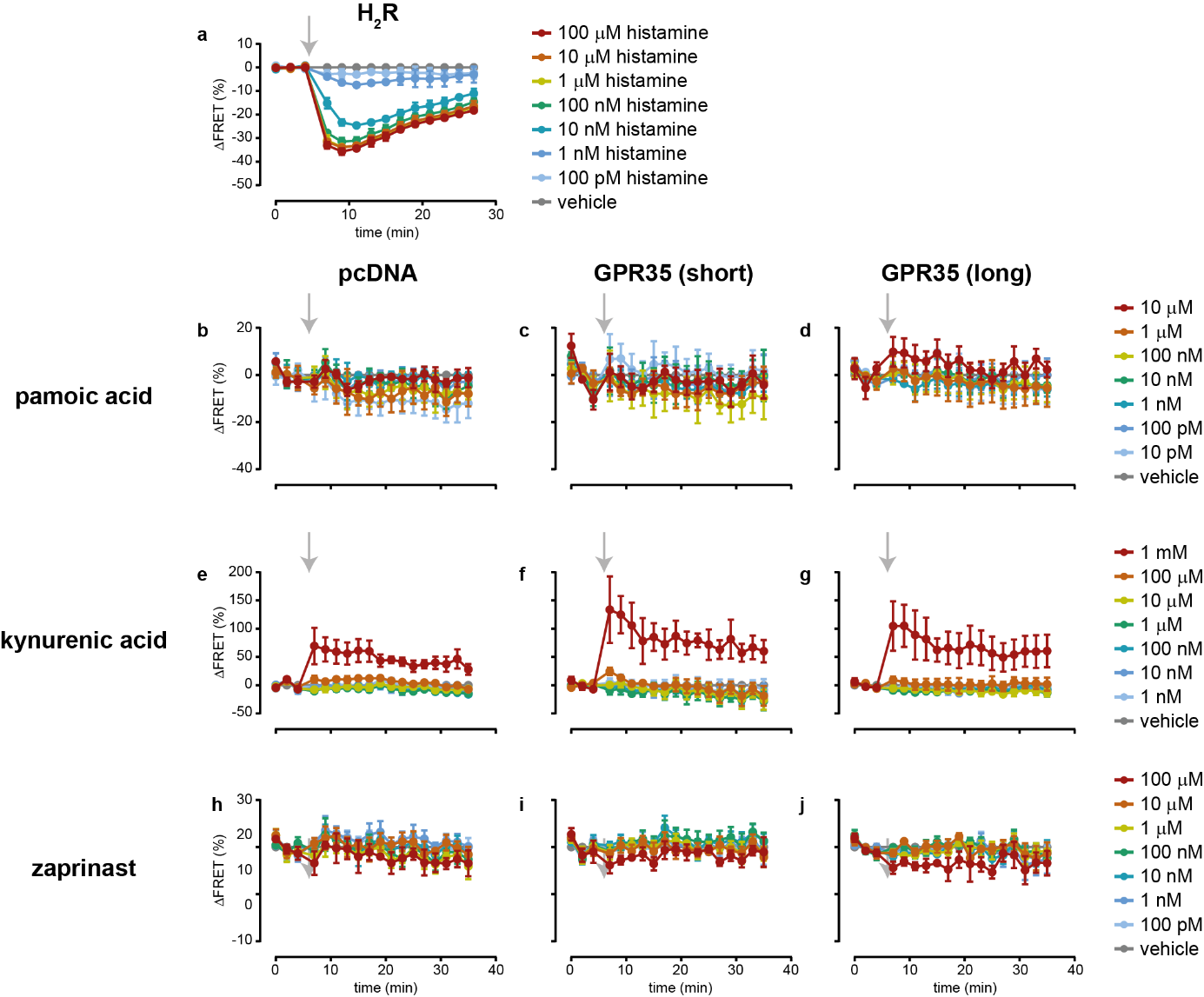


**Figure S10: cAMP FRET sensor time courses. a)** Histamine H_2_ receptor (H_2_R)-mediated increases in FRET following stimulation with histamine. **b-j)** FRET changes induced by pamoic acid (b-d), kynurenic acid (e-g) or zaprinast (h-j) in cells co-transfected with the cAMP FRET sensor and either pcDNA or GPR35 isoforms. All experiments were conducted in HEK293A cells pre-incubated with 5 μM of the phosphodiesterase inhibitor IBMX for five minutes. Data represents mean ± SEM of three independent experiments. The grey arrow indicates the time point ligand or vehicle addition.

**
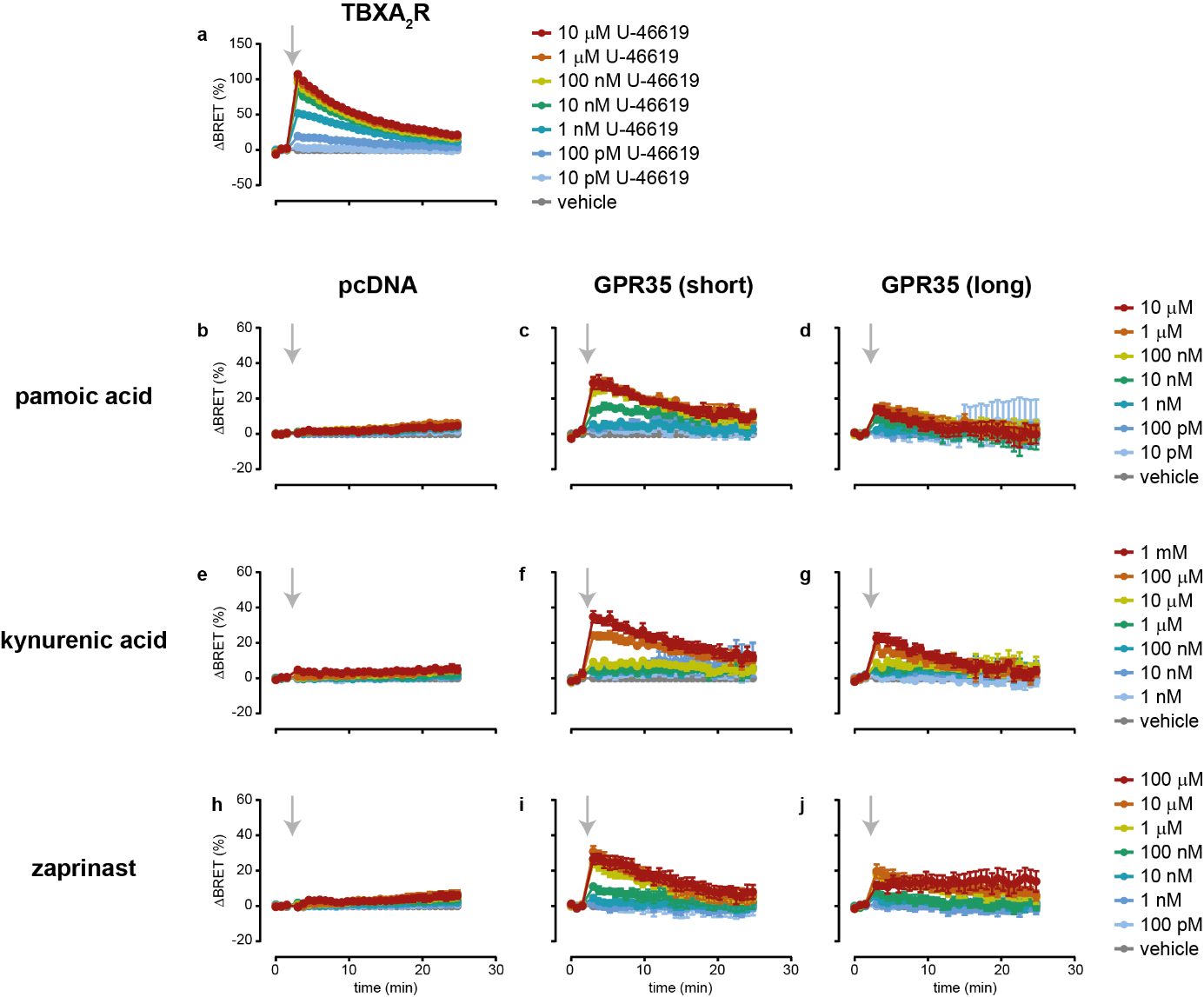
**

**Figure S11: PKN-RBD recruitment time courses. a)** Thromboxane A_2_ receptor (TBXA_2_R)-mediated increases in BRET following stimulation with U-46619. **b-j)** BRET changes induced by pamoic acid (b-d), kynurenic acid (e-g) or zaprinast (h-j) in cells co-transfected with the PKN-RBD recruitment sensor and either pcDNA or GPR35 isoforms. All experiments were conducted in HEK293A cells. Data represents mean ± SEM of three independent experiments. The grey arrow indicates the time point ligand or vehicle addition.


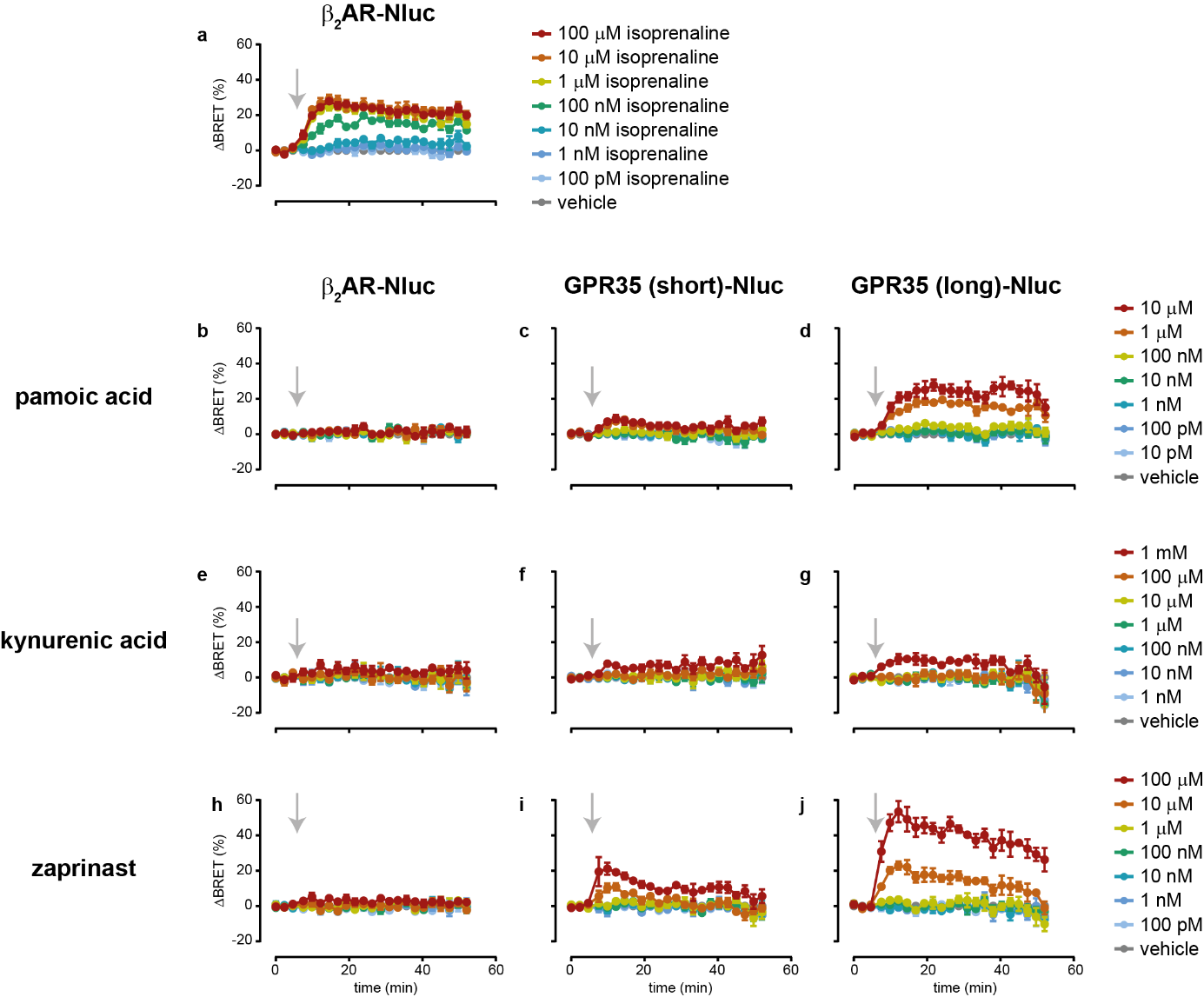


**Figure S12: β-arrestin1 recruitment time courses. a)** Isoprenaline-induced BRET changes between HaloTag-β-arrestin1 to Nluc-tagged β_2_-adrenoceptor (β_2_AR-Nluc). **b-j)** BRET changes induced by pamoic acid (b-d), kynurenic acid (e-g) or zaprinast (h-j) in cells co-transfected with HaloTag-β-arrestin1 and C-terminally Nluc-tagged receptors. All experiments were conducted in HEK293A cells. Data represents mean ± SEM of three to four independent experiments. The grey arrow indicates the time point ligand or vehicle addition.


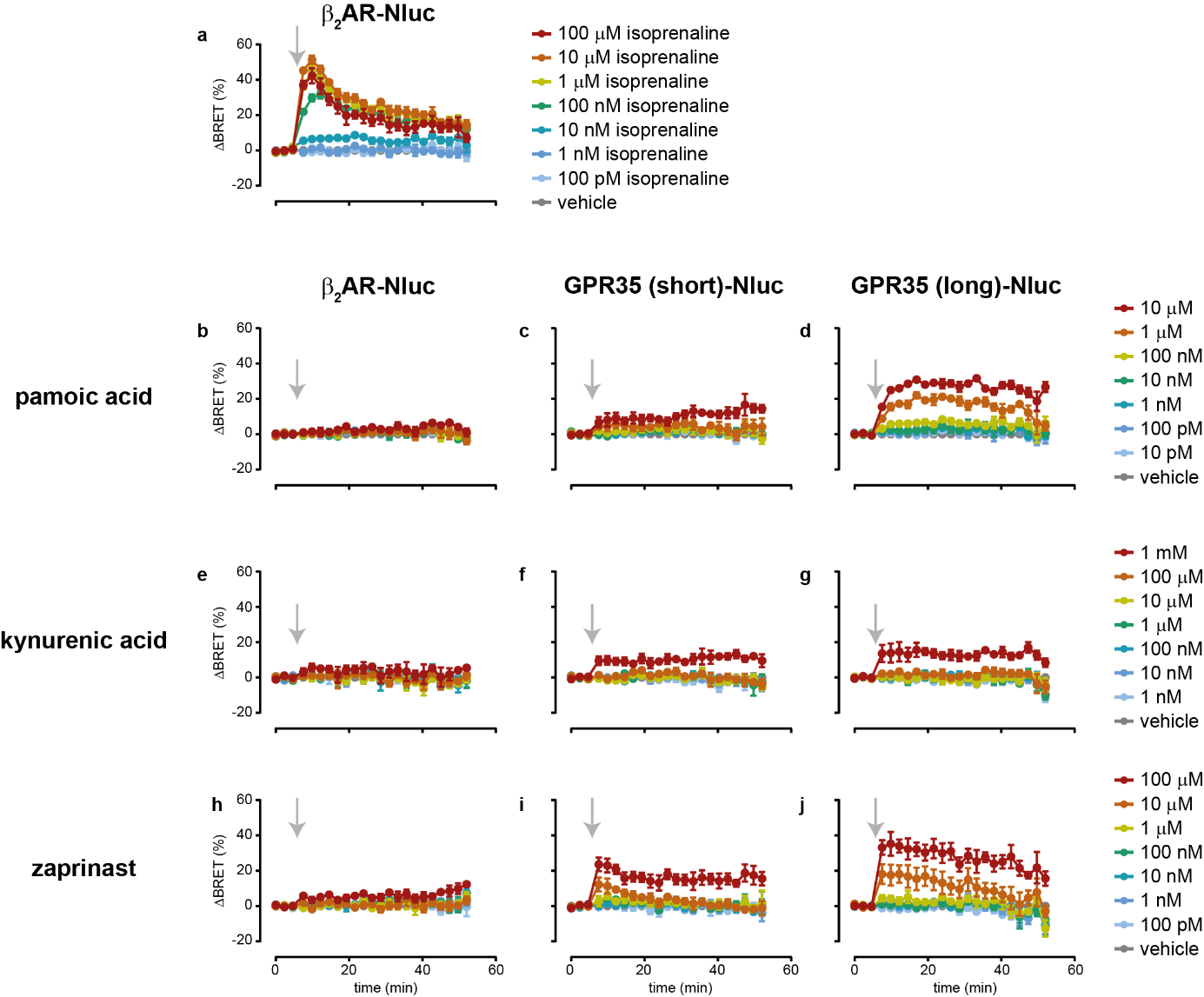


**Figure S13: β-arrestin2 recruitment time courses. a)** Isoprenaline-induced BRET changes between HaloTag-β-arrestin2 to Nluc-tagged β_2_-adrenoceptor (β_2_AR-Nluc). **b-j)** BRET changes induced by pamoic acid (b-d), kynurenic acid (e-g) or zaprinast (h-j) in cells co-transfected with HaloTag-β-arrestin2 and C-terminally Nluc-tagged receptors. All experiments were conducted in HEK293A cells. Data represents mean ± SEM of three to four independent experiments. The grey arrow indicates the time point ligand or vehicle addition.


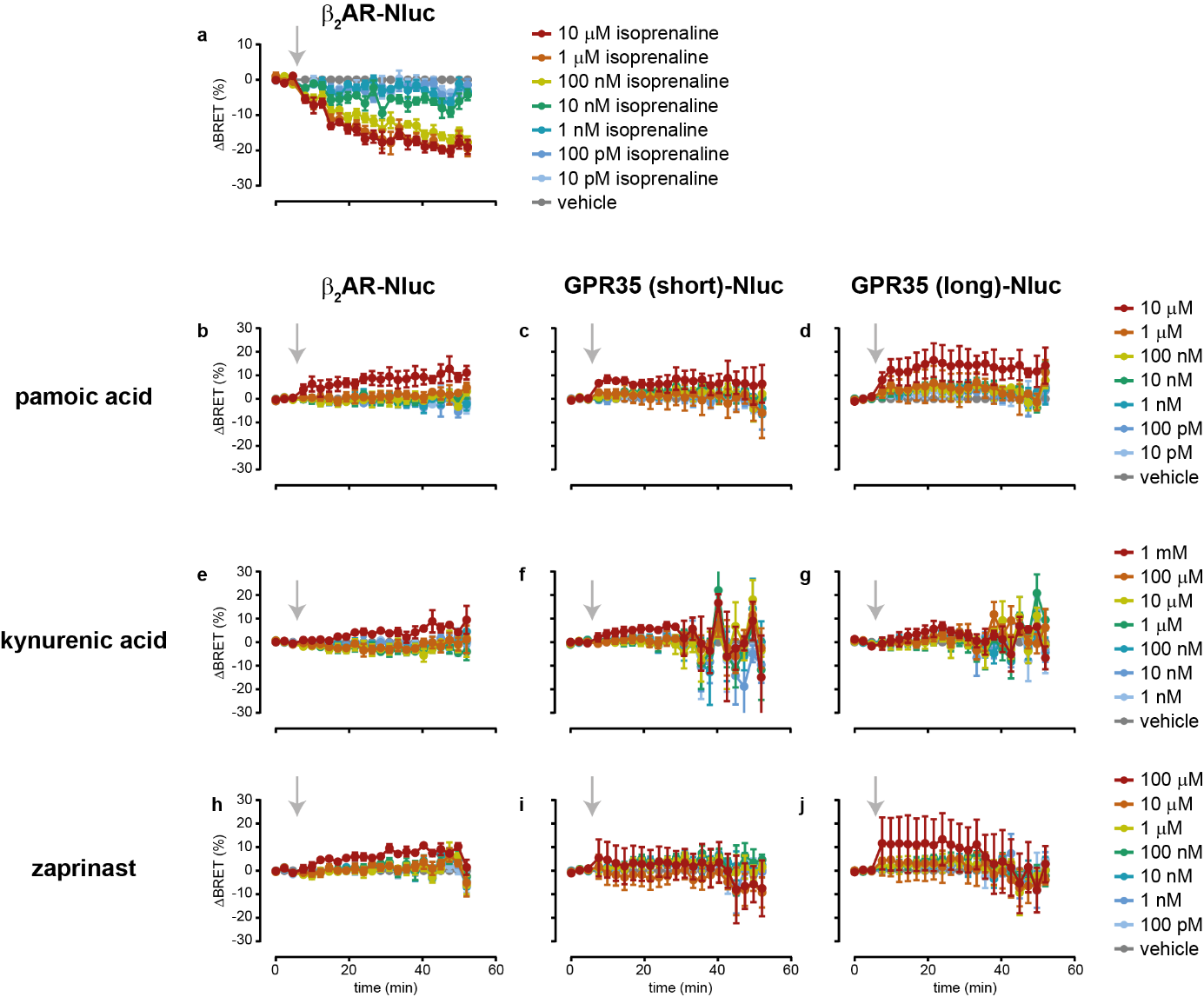


**Figure S14: Receptor internalization time courses. a)** Isoprenaline-induced BRET changes between membrane-anchored HaloTag and Nluc-tagged β_2_-adrenoceptor (β_2_AR-Nluc). **b-j)** BRET changes induced by pamoic acid (b-d), kynurenic acid (e-g) or zaprinast (h-j) in cells co-transfected with membrane-anchored HaloTag and C-terminally Nluc-tagged receptors. All experiments were conducted in HEK293A cells. Data represents mean ± SEM of three to four independent experiments. The grey arrow indicates the time point ligand or vehicle addition.


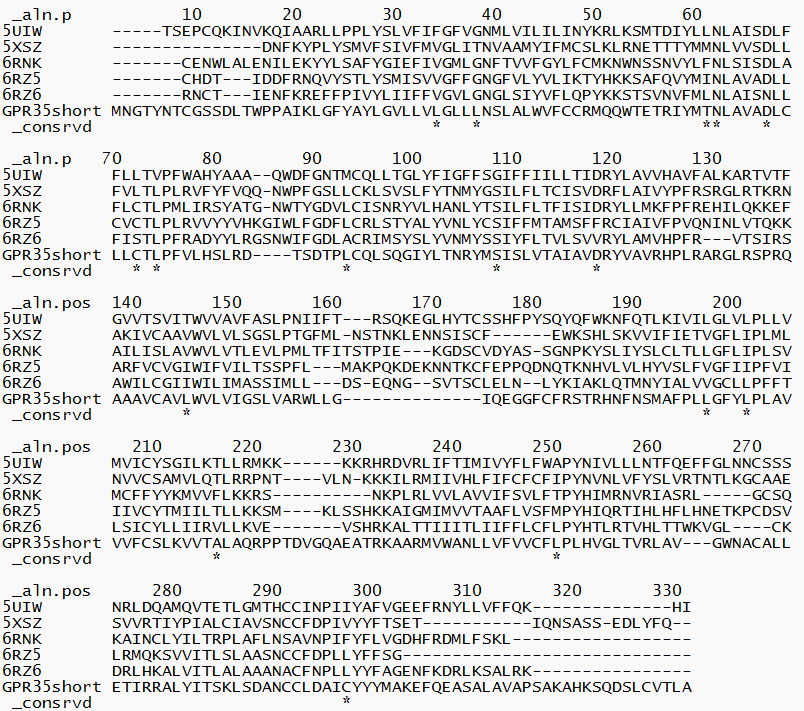


**Figure S15: Sequence alignment of GPR35 short to multiple templates for model building.** The amino acid sequence of GPR35 short was aligned to the sequences of lysophosphatidic acid receptor 6 (LPAR6, PDB 5XSZ), the succinate receptor (SUCNR1, PDB 6RNK) the CC chemokine receptor 5 (CCR5, PDB 5UIW) and the cysteinyl leukotriene receptors 1 (CysLTR1, PDB 6RZ5) and 2 (CysLTR2; PDB 6RZ6) using Modeller10.2.


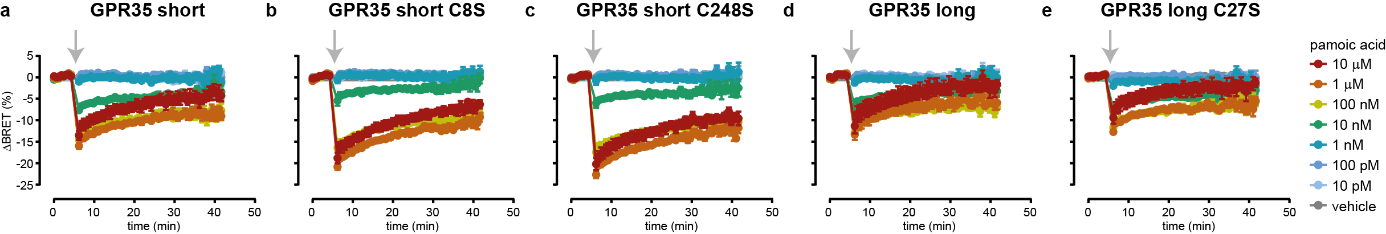


**Figure S16: G_13_ BRET sensor time courses of GPR35 mutants.** The grey arrow indicates the time point of ligand or vehicle addition. All experiments were conducted in HEK293A cells co-transfected with G_13_-CASE and the indicated GPR35 construct/G protein sensor combination. Data represents mean ± SEM of five independent experiments.


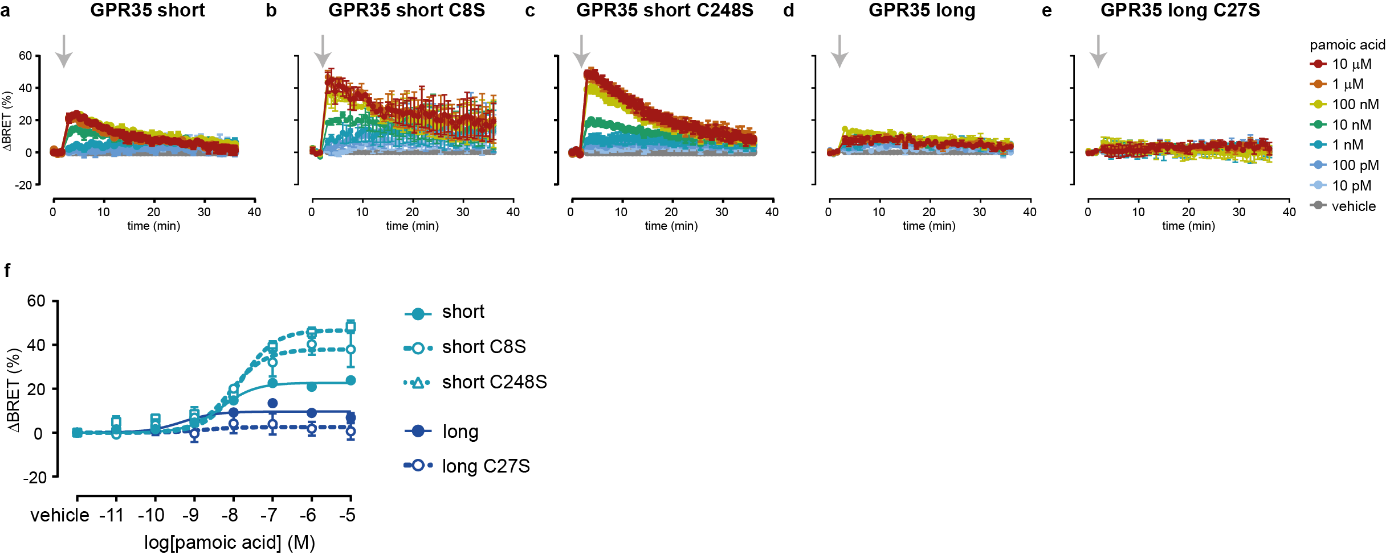


**Figure S17: PKN-RBD recruitment time courses and concentration response curves for GPR35 mutants. a-e)** Time courses of pamoic acid-induced PKN-RBD recruitment. The grey arrow indicates the time point of ligand or vehicle addition. **f)** Corresponding concentration response curves of pamoic acid. All experiments were conducted in HEK293A cells co-transfected with G_13_-CASE and the indicated GPR35 construct/G protein sensor combination. Data represents mean ± SEM of five independent experiments.


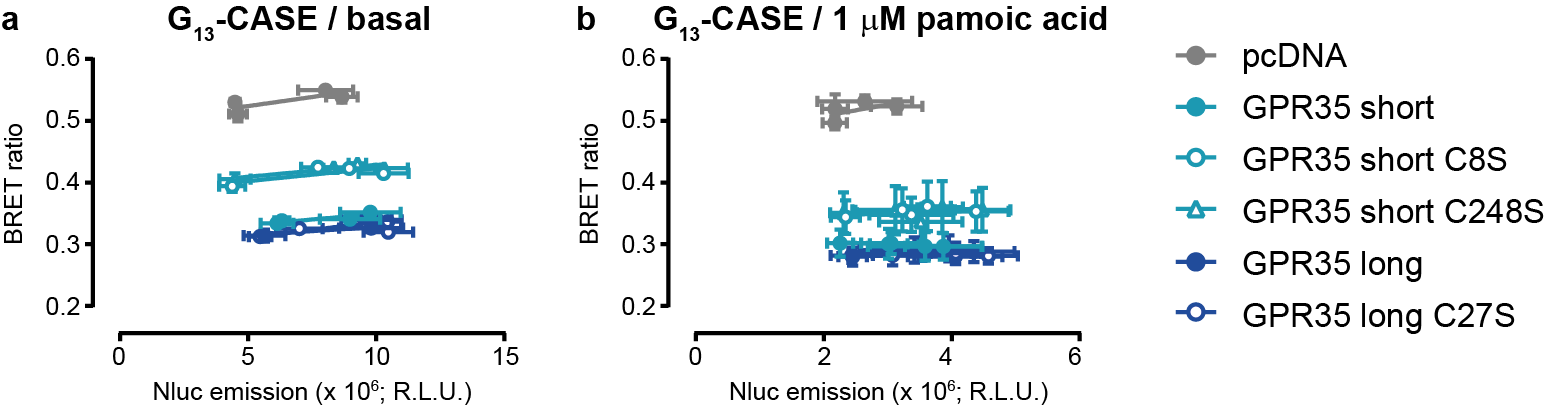


**Figure S18: BRET over Nluc plots for assessment of constitutive G_13_ activation by GPR35 mutants. a)** BRET x Nluc correlation for the indicated GPR35 constructs prior agonist addition. **b)** BRET x Nluc correlation for the indicated GPR35 constructs after pamoic acid addition. Data points were fitted to a linear regression curve to determine BRET_0_ values presented in Fig. 6d and e. All experiments were conducted in HEK293A cells co-transfected with G_13_-CASE and the indicated GPR35 construct. Data represents mean ± SD of four to five independent experiments.


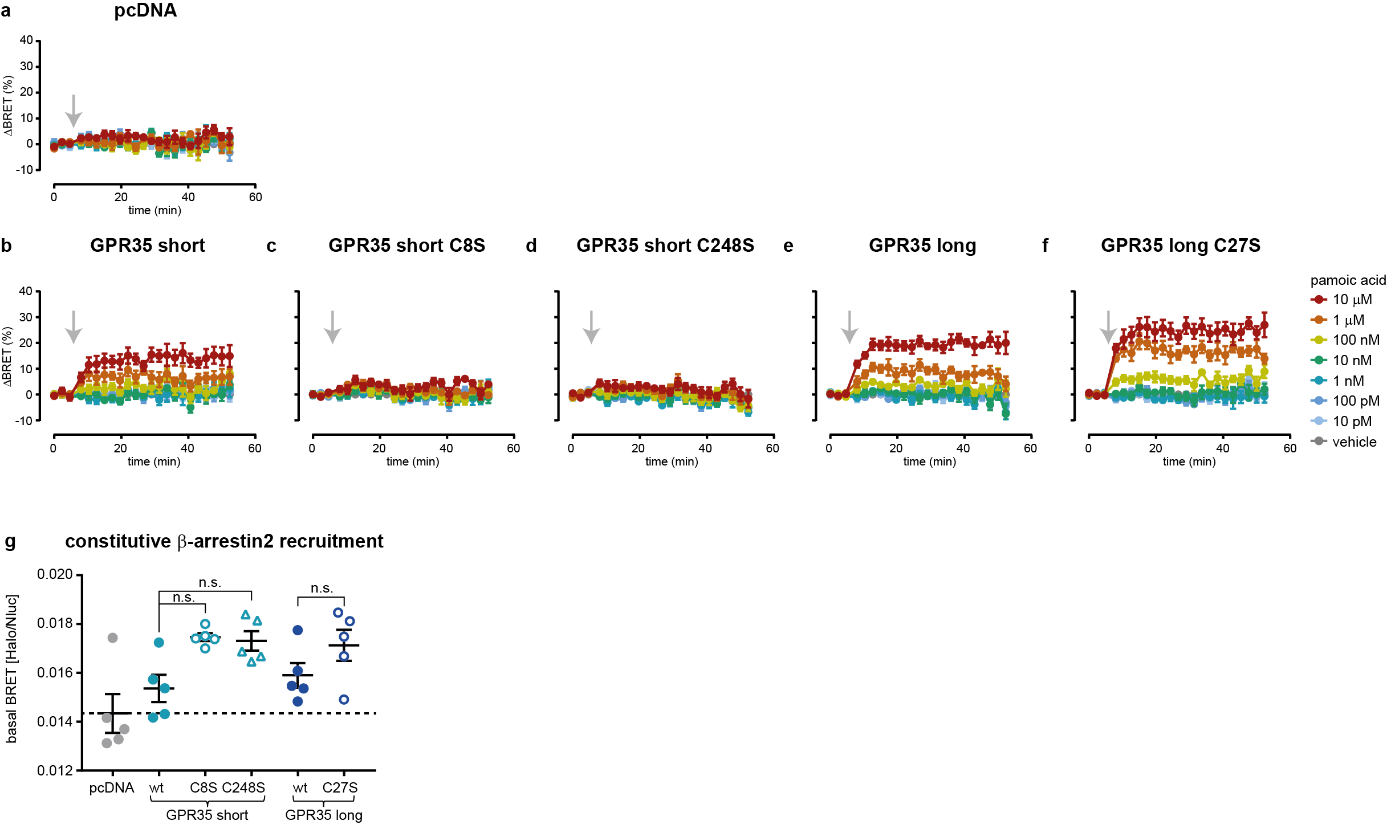


**Figure S19: β-arrestin2 recruitment time courses and basal BRET values for GPR35 mutants. a-f)** Pamoic acid-induced BRET changes between Nluc-β-arrestin2 and membrane-anchored HaloTag fluorescent ligand. The grey arrow indicates the time point of ligand or vehicle addition. g) Corresponding basal BRET values detected prior agonist addition. All experiments were conducted in HEK293A cells co-transfected with Nluc-β-arrestin2, membrane-anchored HaloTag and the indicated GPR35 construct. Data represents mean ± SEM of five independent experiments. Statistical significance in (g) was tested using One-way ANOVA followed by Tukey’s multiple comparison; p < 0.5.

**table S1: EC_50_ values of pamoic acid and maximum ΔFRET/BRET responses at vector-, GPR35 short- or GPR35 long-transfected cells determined with different pharmacological assays.**

| assay | pcDNA | | GPR35 short | | GPR35 long | |
| --- | --- | --- | --- | --- | --- | --- |
|  | % ΔFRET/BRET (mean ± SEM) | pEC_50_ (mean ± SEM) | % ΔFRET/BRET (mean ± SEM) | pEC_50_ (mean ± SEM) | % ΔFRET/BRET (mean ± SEM) | pEC_50_ (mean ± SEM) |
| G_i1_ dissociation | 12.36 ± 17.84 | 4.89 ± 1.12 | 8.64 ± 10.41 | 5.02 ± 1.06 | 6.74 ± 2.06 | 5.33 ± 0.38 |
| G_i2_ dissociation | n.d. | | 8.59 ± 5.24 | 5.20 ± 0.64 | 10.24 ± 10.71 | 10.24 ± 10.71 |
| G_i3_ dissociation | n.d. | | n.d. | | 6.16 ± 3.67 | 6.16 ± 3.67 |
| G_o1_ dissociation | 9.36 ± 17.34 | 4.75 ± 1.42 | 1.03 ± 0.19 | 10.07 ± 0.92 | 0.14 ± 0.49 | 6.82 ± 2.02 |
| G_z_ dissociation | -0.71 ± 0.25 | 9.08 ± 1.30 | -0.69 ± 0.30 | 8.14 ± 1.55 | 0.30 ± 0.21 | 10.50 ± 2.01 |
| G_q_ dissociation | n.d. | | 8.51 ± 18.37 | 4.71 ± 1.40 | 2.56 ± 1.51 | 2.56 ± 1.51 |
| G_15_ dissociation | n.d. | | -1.18 ± 0.32 | 7.29 ± 0.66 | -1.58 ± 0.27 | -1.58 ± 0.27 |
| G_s_ dissociation | 3.38 ± 0.68 | 5.80 ± 0.32 | 4.98 ± 4.83 | 5.18 ± 0.98 | 1.73 ± 1.65 | 5.64 ± 1.02 |
| G_12_ dissociation | -0.20 ± 0.20 | 10.70 ± 3.66 | -9.00 ± 0.34***** | 7.87 ± 0.11 | -6.27 ± 0.17***** | 7.98 ± 0.08 |
| G_13_ dissociation | 11.90 ± 36.23 | 4.56 ± 1.78 | -11.89 ± 0.24***** | 7.96 ± 0.06***** | -8.63 ± 0.20***** | 8.19 ± 0.07***** |
| cAMP reduction | 0.41 ± 2.14 | 10.04 ± 4.06 | -0.89 ± 1.72 | 8.36 ± 2.62 | 2.97 ± 2.52 | 8.54 ± 4.06 |
| p63RhoGEF recruitment | 3.73 ± 0.86 | 8.48 ± 1.36 | 1.74 ± 1.69 | 10.52 ± 2.44 | 0.65 ± 1.33 | 9.33 ± 3.34 |
| DAG generation | 2.46 ± 0.19 | 10.76 ± 0.40 | 1.86 ± 0.19 | 10.48 ± 0.52 | 3.08 ± 0.24 | 8.91 ± 0.40 |
| cAMP generation | -7.06 ± 3.80 | 7.93 ± 6.36 | -5.82 ± 3.05 | 7.67 ± 1.79 | n.d. | |
| PKN-RBD recruitment | 0.79 ± 0.22 | 10.13 ± 1.70 | 28.83 ± 1.46***** | 7.81 ± 0.16 | 14.29 ± 1.05***** | 8.13 ± 0.23 |
| β-arrestin1 recruitment | 0.61 ± 0.69 | 9.08 ± 3.50 | 7.04 ± 0.79***** | 6.46 ± 0.23 | 26.05 ± 2.14***** | 6.38 ± 0.18 |
| β-arrestin2 recruitment | 1.49 ± 0.35 | 10.87 ± 1.12 | 8.78 ± 1.35***** | 6.21 ± 0.39 | 29.38 ± 1.57***** | 6.39 ± 0.12 |
| receptor internalization | 28.03 ± 104.3 | 4.70 ± 2.35 | n.d. | | 34.10 ± 154.10 | 4.72 ± 3.30 |

n.d.: Fit considered “ambiguous”, “interrupted” or “not converged” by GraphPad Prism or mean and error values only estimated (“~”). All other values calculated by GraphPad Prism are provided in the table even if these were only extrapolated because no saturation of the response was reached with the tested ligand concentrations.
Statistical differences between pEC_50_ and ΔFRET/BRET values have only been assessed if the nonlinear fits for GPR35 short and long deviated from the pcDNA fit according to extra-sum-of-squares F-test ‘One curve for all datasets’; p < 0.05.
*****: statistically different compared to the other GPR35 isoform according to an extra-sum-of-squares F-test; p < 0.05. Absence of “*” indicates that the test has been performed but did not yield statistical difference.

**table S2: EC_50_ values of kynurenic acid and maximum ΔFRET/BRET responses at vector-, GPR35 short- or GPR35 long-transfected cells determined with different pharmacological assays.**

| assay | pcDNA | | GPR35 short | | GPR35 long | |
| --- | --- | --- | --- | --- | --- | --- |
|  | % ΔFRET/BRET (mean ± SEM) | pEC_50_ (mean ± SEM) | % ΔFRET/BRET (mean ± SEM) | pEC_50_ (mean ± SEM) | % ΔFRET/BRET (mean ± SEM) | pEC_50_  (mean ± SEM) |
| G_i1_ dissociation | 15.01 ± 4.99 | 2.80 ± 0.35 | 20.52 ± 7.03 | 2.57 ± 0.29 | 20.26 ± 7.79 | 2.51 ± 0.32 |
| G_i2_ dissociation | 12.24 ± 3.68 | 2.81 ± 0.30 | 19.39 ± 7.60 | 2.52 ± 0.31 | 19.52 ± 8.49 | 2.45 ± 0.33 |
| G_i3_ dissociation | 35.68 ± 42.35 | 1.99 ± 0.66 | 24.57 ± 10.12 | 2.27 ± 0.27 | 62.51 ± 106.10 | 1.69 ± 0.85 |
| G_o1_ dissociation | n.d. | | 1.04 ± 0.20 | 7.61 ± 1.16 | 2.41 ± 5.28 | 3.03 ± 2.83 |
| G_z_ dissociation | 0.98 ± 0.93 | 3.48 ± 1.15 | n.d. | | 7.03 ± 42.38 | 2.45 ± 3.40 |
| G_q_ dissociation | 31.62 ± 36.78 | 1.97 ± 0.64 | 67.96 ± 206.90 | 1.53 ± 1.45 | 18.92 ± 22.33 | 2.13 ± 0.69 |
| G_15_ dissociation | n.d. | | 1.46 ± 0.31 | 3.89 ± 0.40 | n.d. | |
| G_s_ dissociation | n.d. | | n.d. | | n.d. | |
| G_12_ dissociation | n.d. | | -7.81 ± 0.64***** | 4.39 ± 0.16 | -4.95 ± 0.50***** | 4.20 ± 0.17 |
| G_13_ dissociation | 0.50 ± 0.14 | 5.43 ± 1.12 | -10.07 ± 0.52***** | 4.13 ± 0.09***** | -7.01 ± 0.46***** | 4.59 ± 0.14***** |
| cAMP reduction | n.d. | | n.d. | | n.d. | |
| p63RhoGEF recruitment | 4.13 ± 23.39 | 3.15 ± 4.43 | n.d. | | -1.27 ± 2.66 | 6.17 ± 4.56 |
| DAG generation | 16.55 ± 14.52 | 3.23 ± 0.94 | 24.28 ± 16.74 | 3.07 ± 0.62 | 17.50 ± 6.97 | 3.23 ± 0.43 |
| cAMP generation | n.d. | | n.d. | | n.d. | |
| PKN-RBD recruitment | 8.48 ± 5.50 | 3.08 ± 0.61 | 36.19 ± 1.93***** | 4.27 ± 0.12 | 22.62 ± 1.74***** | 4.60 ± 0.20 |
| β-arrestin1 recruitment | 10.31 ± 63.30 | 2.75 ± 3.61 | 6.69 ± 2.90 | 3.38 ± 0.50 | n.d. | |
| β-arrestin2 recruitment | 2.36 ± 0.81 | 7.90 ± 1.42 | n.d. | | 33.07 ± 48.66 | 2.85 ± 1.04 |
| receptor internalization | n.d. | | n.d. | | n.d. | |

n.d.: Fit considered “ambiguous”, “interrupted” or “not converged” by GraphPad Prism or mean and error values only estimated (“~”). All other values calculated by GraphPad Prism are provided in the table even if these were only extrapolated because no saturation of the response was reached with the tested ligand concentrations.
Statistical differences between pEC_50_ and ΔFRET/BRET values have only been assessed if the nonlinear fits for GPR35 short and long deviated from the pcDNA fit according to extra-sum-of-squares F-test ‘One curve for all datasets’; p < 0.05.
*****: statistically different compared to the other GPR35 isoform according to an extra-sum-of-squares F-test; p < 0.05. Absence of “*” indicates that the test has been performed but did not yield statistical difference.

**table S3: EC_50_ values of zaprinast and maximum ΔFRET/BRET responses at vector-, GPR35 short- or GPR35 long-transfected cells determined with different pharmacological assays.**

| assay | pcDNA | | GPR35 short | | GPR35 long | |
| --- | --- | --- | --- | --- | --- | --- |
|  | % ΔFRET/BRET (mean ± SEM) | pEC_50_ (mean ± SEM) | % ΔFRET/BRET (mean ± SEM) | pEC_50_ (mean ± SEM) | % ΔFRET/BRET (mean ± SEM) | pEC_50_ (mean ± SEM) |
| G_i1_ dissociation | 2.65 ± 0.74 | 4.64 ± 0.55 | 0.98 ± 0.13 | 10.24 ± 0.74 | 4.33 ± 37.43 | 3.64 ± 6.05 |
| G_i2_ dissociation | 3.31 ± 3.88 | 4.41 ± 1.51 | 1.54 ± 0.39 | 7.00 ± 0.83 | n.d. | |
| G_i3_ dissociation | 1.07 ± 0.26 | 8.01 ± 1.20 | 1.23 ± 0.11 | 9.74 ± 0.47 | n.d. | |
| G_o1_ dissociation | n.d. | | -1.97 ± 0.57 | 4.87 ± 0.37 | -3.05 ± 0.73 | 4.90 ± 0.33 |
| G_z_ dissociation | n.d. | | n.d. | | n.d. | |
| G_q_ dissociation | n.d. | | -0.24 ± 0.34 | 7.39 ± 5.35 | -0.32 ± 0.30 | 6.94 ± 1.14 |
| G_15_ dissociation | 0.21 ± 0.10 | 9.36 ± 1.60 | -3.02 ± 0.29 | 4.87 ± 0.15 | -3.94 ± 0.47 | 4.85 ± 0.19 |
| G_s_ dissociation | 0.51 ± 0.28 | 8.37 ± 2.51 | -0.39 ± 0.25 | 9.89 ± 2.89 | 0.23 ± 0.32 | 6.75 ± 2.45 |
| G_12_ dissociation | n.d. | | -8.20 ± 0.27***** | 7.10 ± 0.10 | -5.38 ± 0.21***** | 7.11 ± 0.11 |
| G_13_ dissociation | -1.02 ± 0.18 | 10.45 ± 1.13 | -12.93 ± 0.19***** | 6.72 ± 0.04***** | -9.71 ± 0.14***** | 6.86 ± 0.04***** |
| cAMP reduction | -11.66 ± 5.70 | 5.30 ± 1.14 | n.d. | | 2.65 ± 4.75 | 5.17 ± 3.12 |
| p63RhoGEF recruitment | 1.24 ± 0.40 | 7.94 ± 0.92 | 1.35 ± 1.33 | 7.01 ± 2.58 | 1.30 ± 1.40 | 7.57 ± 4.41 |
| DAG generation | 2.28 ± 0.34 | 6.87 ± 0.59 | 1.91 ± 0.26 | 9.76 ± 0.69 | 2.28 ± 0.26 | 7.39 ± 0.72 |
| cAMP generation | -0.48 ± 1.62 | 7.13 ± 1.69 | 1.33 ± 1.17 | 9.86 ± 3.75 | n.d. | |
| PKN-RBD recruitment | 0.70 ± 0.39 | 9.71 ± 2.67 | 28.68 ± 1.30***** | 6.76 ± 0.14 | 16.47 ± 1.49***** | 7.19 ± 0.30 |
| β-arrestin1 recruitment | -0.13 ± 0.80 | 8.55 ± 2.41 | 20.88 ± 1.62***** | 5.03 ± 0.14 | 62.67 ± 3.87***** | 4.76 ± 0.10 |
| β-arrestin2 recruitment | n.d. | | 24.27 ± 2.60***** | 4.74 ± 0.17 | 37.52 ± 3.08***** | 4.98 ± 0.14 |
| receptor internalization | 6.48 ± 1.89 | 4.70 ± 0.49 | 1.07 ± 2.66 | 5.66 ± 3.71 | 14.53 ± 18.55 | 4.35 ± 2.01 |

n.d.: Fit considered “ambiguous”, “interrupted” or “not converged” by GraphPad Prism or mean and error values only estimated (“~”). All other values calculated by GraphPad Prism are provided in the table even if these were only extrapolated because no saturation of the response was reached with the tested ligand concentrations.
Statistical differences between pEC_50_ and ΔFRET/BRET values have only been assessed if the nonlinear fits for GPR35 short and long deviated from the pcDNA fit according to extra-sum-of-squares F-test ‘One curve for all datasets’; p < 0.05.
*****: statistically different compared to the other GPR35 isoform according to an extra-sum-of-squares F-test; p < 0.05. Absence of “*” indicates that the test has been performed but did not yield statistical difference.

**table S4: EC_50_ values** **and maximum ΔFRET/BRET responses of pamoic acid at GPR35 point mutants.**

| assay | GPR35 short wt | | GPR35 short C8S | | GPR35 short C248S | | GPR35 long wt | | GPR35 C27S | |
| --- | --- | --- | --- | --- | --- | --- | --- | --- | --- | --- |
|  | % ΔBRET  (mean ± SEM) | pEC_50_ (mean ± SEM) | % ΔBRET  (mean ± SEM) | pEC_50_ (mean ± SEM) | % ΔBRET  (mean ± SEM) | pEC_50_ (mean ± SEM) | % ΔBRET  (mean ± SEM) | pEC_50_ (mean ± SEM) | % ΔBRET  (mean ± SEM) | pEC_50_ (mean ± SEM) |
| G_13_ dissociation | -13.20 ± 0.40 | 8.06 ± 0.09 | -17.97 ± 0.48***** | 7.61 ± 0.07***** | -19.76 ± 0.46***** | 7.62 ± 0.07***** | -10.18 ± 0.57 | 8.15 ± 0.16 | -9.49 ± 0.45 | 8.35 ± 0.15 |
| PKN-RBD recruitment | 22.74 ± 0.74 | 8.32 ± 0.10 | 37.98 ± 2.40***** | 8.07 ± 0.18 | 46.71 ± 1.90***** | 7.82 ± 0.11***** | 9.64 ± 0.79 | 9.33 ± 0.28 | 2.57 ± 1.64***** | 8.76 ± 2.01 |
| β-arrestin2 recruitment | 13.95 ± 2.20 | 6.17 ± 0.28 | 3.36 ± 0.66***** | 7.97 ± 0.73 | 3.17 ± 0.84***** | 7.46 ± 0.76 | 21.01 ± 1.67 | 5.94 ± 0.14 | 27.22 ± 2.01***** | 6.46 ± 0.17***** |

*****: statistically different compared to the respective wildtype GPR35 isoform according to an extra-sum-of-squares F-test; p < 0.05. Absence of “*” indicates that the test has been performed but did not yield statistical difference.
